# A Mathematical Model of the Mouse Atrial Myocyte With Inter-Atrial Electrophysiological Heterogeneity

**DOI:** 10.3389/fphys.2020.00972

**Published:** 2020-08-06

**Authors:** Henggui Zhang, Shanzhuo Zhang, Wei Wang, Kuanquan Wang, Weijian Shen

**Affiliations:** ^1^Department of Physics and Astronomy, Biological Physics Group, School of Physics & Astronomy, The University of Manchester, Manchester, United Kingdom; ^2^Peng Cheng Laboratory, Shenzhen, China; ^3^School of Computer Science and Technology, Harbin Institute of Technology, Harbin, China; ^4^Shenzhen Key Laboratory of Visual Object Detection and Recognition, Harbin Institute of Technology, Shenzhen, China

**Keywords:** mouse atrial, action potential, mathematical model, action potential, Ca^2+^ handling, atrial heterogeneity, signaling pathway

## Abstract

Biophysically detailed mathematical models of cardiac electrophysiology provide an alternative to experimental approaches for investigating possible ionic mechanisms underlying the genesis of electrical action potentials and their propagation through the heart. The aim of this study was to develop a biophysically detailed mathematical model of the action potentials of mouse atrial myocytes, a popular experimental model for elucidating molecular and cellular mechanisms of arrhythmogenesis. Based on experimental data from isolated mouse atrial cardiomyocytes, a set of mathematical equations for describing the biophysical properties of membrane ion channel currents, intracellular Ca^2+^ handling, and Ca^2+^-calmodulin activated protein kinase II and β-adrenergic signaling pathways were developed. Wherever possible, membrane ion channel currents were modeled using Markov chain formalisms, allowing detailed representation of channel kinetics. The model also considered heterogeneous electrophysiological properties between the left and the right atrial cardiomyocytes. The developed model was validated by its ability to reproduce the characteristics of action potentials and Ca^2+^ transients, matching quantitatively to experimental data. Using the model, the functional roles of four K^+^ channel currents in atrial action potential were evaluated by channel block simulations, results of which were quantitatively in agreement with existent experimental data. To conclude, this newly developed model of mouse atrial cardiomyocytes provides a powerful tool for investigating possible ion channel mechanisms of atrial electrical activity at the cellular level and can be further used to investigate mechanisms underlying atrial arrhythmogenesis.

## Introduction

Murine hearts are commonly used as animal models in cardiac research for investigating possible molecular and cellular bases of cardiac arrhythmias (Martin et al., 2012; Huang, 2016). Over years, plethora experimental data on cardiac electrophysiology of the mouse heart have been obtained in a wide range of physiological and pathological conditions. Now it is a challenge to integrate these experimental data obtained at different scales into an integrated mathematical model for systematically elucidating better functional roles of ion channels in atrial electrical excitations and arrhythmogenesis. Up to date, a number of mathematical models have been developed for cellular action potentials (APs) of different types of mouse cardiomyocytes. Examples include the first mathematical model of mouse sinoatrial node by Mangoni et al. (2006), which was further updated by incorporating biophysical properties of membrane ionic currents and intracellular Ca^2+^ handling mechanisms by Kharche et al. (2011), the first mouse ventricular cell models developed by Bondarenko et al. (2004), which were updated by incorporating newer experimental data by Li et al. (2010). Recently, mouse ventricular cell models with β-adrenergic signaling regulation and with calmodulin (CaM) mediating Ca^2+^-dependent regulation were developed by Yang and Saucerman (2012) and Morotti et al. (2014), respectively. These models provide useful tools for underpinning insights into the regulation of Ca^2+^ handling in physiological and pathological conditions.

However, experimental data of mouse atrial electrophysiology is spare due to its limited use for the study of atrial fibrillation, as the mouse heart is too small to initiate and maintain atrial arrhythmias (Huang, 2016) in normal conditions. Recently, however, sustained atrial tachycardia and fibrillation in mouse heart has been reported (Wakimoto et al., 2001), and intensely investigated with transgenic or wild type mice (Kovoor et al., 2001; Verheule et al., 2004; Chelu et al., 2009; Choi et al., 2012). These experimental data provide sufficient foundation to develop a mouse atrial cell model. In addition, after decades of experimental studies on the intracellular signaling network (Yang and Saucerman, 2011), sufficient data are also available to construct a biophysically detailed model of mouse atrial myocytes, with consideration of intracellular signaling pathways to simulate the regulation from nervous and endocrine systems (Ramos-Franco et al., 1998; Doi et al., 2005).

Furthermore, electrophysiological heterogeneity within or between the mouse left and right atria has been implicated as a factor initiating and/or maintaining atrial rhythm disturbances (Nygren et al., 2004). Such electrical heterogeneity between left and right mouse atria has also been found in many studies (Lomax et al., 2003a; Trépanier-Boulay et al., 2004; Hu et al., 2005; Nakamura et al., 2010), in which left-to-right differences in several K^+^ currents, as well as in the action potential duration (APD) of isolated atrial myocytes, were reported. A mouse atrial cell model considering the heterogeneity between left and right atria will be helpful on the study of the genesis of AF, such as to investigate how the fast rotors in the left atrium (LA) propagate toward the right atrium (RA) (Sarmast et al., 2003).

The main goal of this study is to develop a mathematical model of mouse atrial myocytes to simulate mouse atrial APs and intracellular Ca^2+^ handling mechanisms. In the developed model, Markov chain formalizations were used to describe membrane ion channels for three main potassium currents (the transient outward K^+^ current (I_to_), the ultra-rapidly activating delayed rectifier K^+^ current (I_Kur_) and the rapid delayed rectifier K^+^ current (I_Kr_) and the L-type Ca^2 +^ channels (LTCCs). Heterogeneity between left and right atria was considered to derive a left atrial cell model based on the baseline model of right atria. The characteristics of AP and Ca^2+^ transient (CaT) generated by our model were compared with mouse atrial experimental findings, which validated the model development. The Ca^2+^-calmodulin activated protein kinase II (CaMKII) and β-adrenergic signaling pathways were also included.

## Model Development

Details of model implementation, pacing protocols, and performance of the model can be found in Supplementary Document 1. The source code in C++ of the model is available under the request to the authors (henggui.zhang@manchester.ac.uk).

### General Structure

**FIGURE 1:**
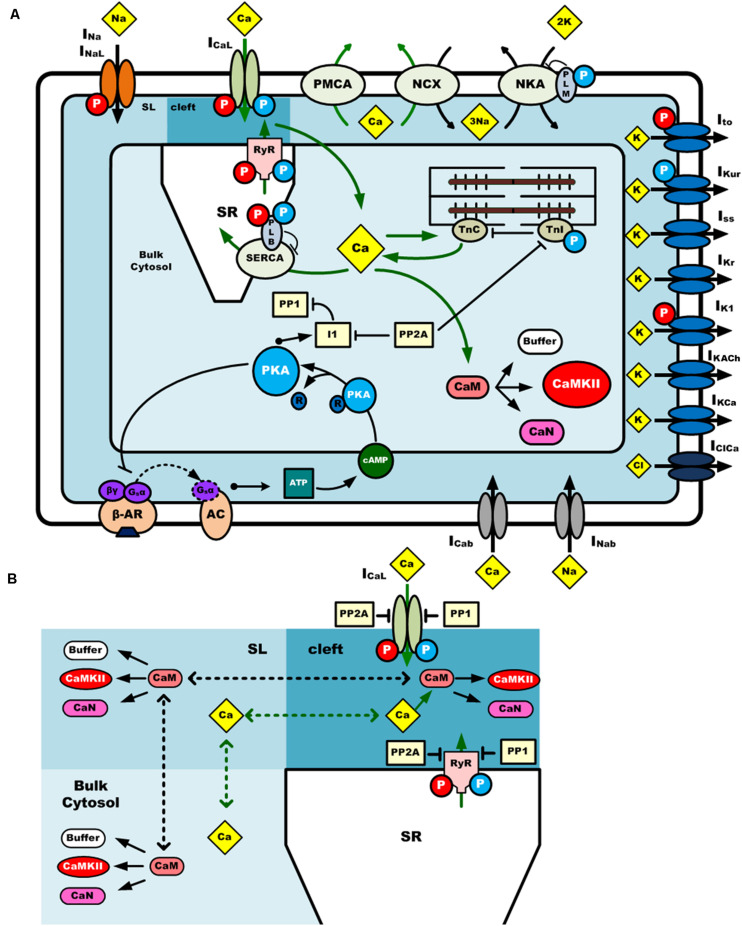
A schematic diagram of the computational model of a mouse atrial myocyte. **(A)** Ionic channel currents, fluxes, signaling pathways and physical compartments of the mouse atrial model. **(B)** Diffusion of Ca^2+^ and CaM in three compartments. CaMKII is activated by Ca^2+^-CaM binding in the dyadic cleft, sub-sarcolemma area (SL), and bulk cytosol. Phosphatases 1 and phosphatases 2A oppose phosphorylation by either kinase. Ca^2+^ and CaM can diffuse across these three regions at different diffusion coefficients. The blue and red circle with “P” in it indicates the phosphorylation of the target by PKA or CaMKII, respectively. I_Na_, fast Na^+^ current; I_NaL_, late Na^+^ current; I_CaL_ (or LTCC), L-type Ca^2+^ current; I_to_, transient outward K^+^ current; I_Kur_, ultra-rapidly activating delayed rectifier K^+^ current; I_Kr_, rapid delayed rectifier K^+^ current; I_ss_, non-inactivating steady-state voltage-activated K^+^ current; I_K1_, time-independent inwardly rectifying K^+^ current; I_KACh_, acetylcholine-activated K^+^ current; I_KCa_, small conductance Ca^2+^-activated K^+^ current; I_Nab_, background Na^+^ current; I_Cab_, background Ca^2+^ current; I_Kb_, background K^+^ current; I_Clb_, background Cl^–^ current; I_ClCa_, Ca^2+^-activated Cl^–^ current; PMCA, plasma membrane Ca^2+^ pump; NKA, Na^+^/K^+^-ATPase; NCX, Na^+^/Ca^2+^ exchanger.

The mouse atrial myocyte model was developed based on the basal model of the mouse ventricular cell model of Morotti et al. (2014) (body temperature), which was extensively modified to match experimental data of mouse atrial cells. The main changes to the original model are described below, and more details are summarized in Supplementary Document 1. The model consists of 159 coupled ordinary differential equations, each of which describes kinetic properties of membrane ion channels, pump and exchanger currents, intracellular ionic homeostasis, and the CaMKII and β-adrenergic signaling pathway. To better describe gating kinetics of ionic channels of I_to_, I_Kur_, and I_Kr_, Markov chain models were used. Equations for CaMKII and β-adrenergic signaling pathways are inherited from the Morotti et al. model, with some parameters of the activation of CaMKII and β-adrenergic activation of protein kinase A (PKA), and their phosphorylation on excitation-contraction coupling (ECC) targets, being modified based on atrial experimental findings. A schematic diagram of the mouse atrial myocyte model is shown in Figure 1.

### Modeling of Membrane Currents

#### LTCC

LTCC provides main Ca^2+^ influx for myocytes and acts as a trigger for Ca^2+^ release from the sarcoplasmic reticulum (SR). There are four types of LTCCs: Ca_v1.1_ to Ca_v1.4_, among which Ca_*v1.2*_ plays a major role during cardiac ECC (Hofmann et al., 1999). I_CaL_ has the kinetics of a fast activation and a slower inactivation time course.

**FIGURE 2:**
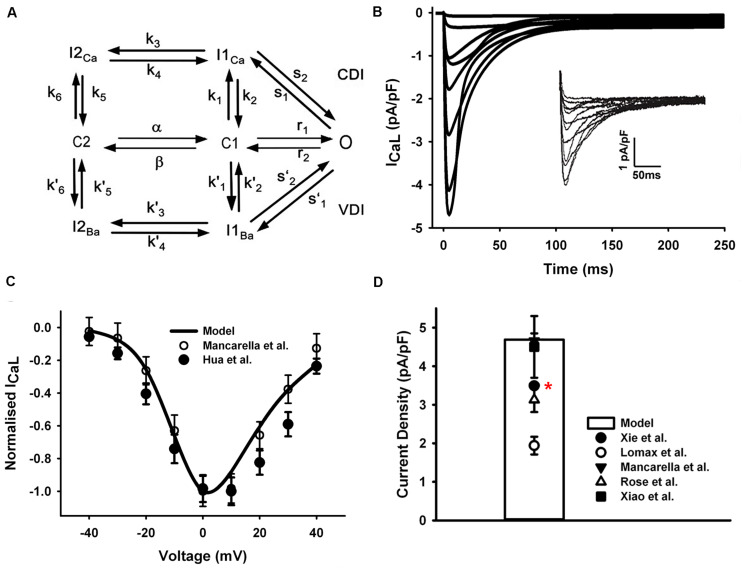
I_CaL_ in the atrial myocyte model and comparison with experimental data. **(A)** A schematic diagram of the Markov chain model of the L-type Ca^2+^ channel (Mahajan et al., 2008). **(B)** Current traces from simulation and experimental records (inset) from Mancarella et al. (2008). **(C)** Normalized I–V relationship of I_CaL_. **(D)** Peak simulated current density compared with experimental data (Rose et al., 1992; Lomax et al., 2003a; Xiao et al., 2006; Mancarella et al., 2008; Xie et al., 2012). All experiments were conducted at room temperature except the data from Xie et al. at body temperature (marked with a red star).

The formulation of LTCC in our model was based on a previous Markov chain model developed by Mahajan et al. (2008) (Figure 2A), with CaMKII and PKA phosphorylation modules being updated for mouse ventricles by Morotti et al. (2014). The LTCC model has a minimum scheme of seven states, which incorporates both calcium-dependent inactivation and voltage-dependent inactivation, where the transitions between C2 and C1 are strongly voltage-dependent, and the transitions from C1 to O are voltage-independent and determine the steady-state open probability.

In order to fit the experimental measures, the voltage dependence of the steady-state transition between C2 and C1 was manually shifted left by 4.4 mV (4.4 mV was added on the voltage in the voltage-dependent transition rates α and β shown in Figure 2A). The current traces shown in Figure 2B were simulated by using the same voltage-clamp protocol as described in Mancarella et al. (2008): the I_CaL_ was activated by a series of 250-ms depolarization pulses from a holding potential of −90 mV, to test potentials ranging from −40 to +40 mV (10-mV steps). And the resultant normalized I–V relationship was in good agreements with experimental observations (Mancarella et al., 2008; Hua et al., 2012) (Figure 2C). The total conductance of I_CaL_ was reduced to 1/4 of the ventricular cell model and the simulated peak current density is 4.76 pA/pF, which is in the range of various mouse atrial experimental data shown in Figure 2D.

#### I_to_

I_to_ is a rapidly activating and inactivating K^+^ current, encoded by K_v4.2_ and K_v4.3_ (Nerbonne, 2000). Xu et al. (1999c) reported that the elimination of I_to_ gave rise to substantial prolonged atrial APD, suggesting that I_to_ plays a prominent role in mouse atrial repolarization.

**FIGURE 3:**
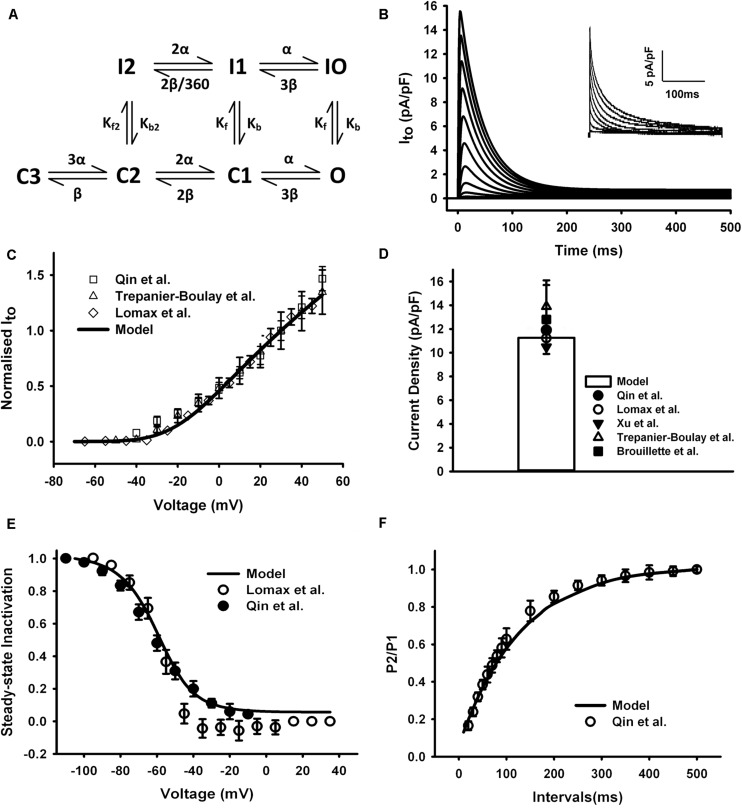
I_to_ in the atrial myocyte model and comparison with experimental data. **(A)** Schematic diagram of seven-state Markov chain model of I_to_ (Campbell et al., 1993). **(B)** Current traces for the corresponding voltage clamp protocol. The inset shows the experimental record. **(C)** I–V relationship, current normalized to its amplitude at +30 mV. **(D)** Current density at +30 mV. **(E)** Steady-state inactivation curve. **(F)** Recovery curve from inactivation. (Experimental data: Lomax et al., 2003a; Trépanier-Boulay et al., 2004; Qin et al., 2012).

The equations for I_to_ in the mouse atrial myocyte were formulated based on the Markov chain model of Campbell et al. (1993) that comprised seven states as schematically shown in Figure 3A. Transition rates were refitted with a non-manual method (details in Supplementary Document 1). Simulated current traces in Figure 3B were similar to those seen in experiments (the inset in Figure 3B). The current traces were obtained by using a protocol of 500-ms voltage steps in 10-mV increments between −70 and +50 mV from a holding potential of −75 mV. The normalized I–V relationship of our model was then calculated and compared with experimental data (Figure 3C). Simulation of I_to_ shows a peak current density of 11.6 pA/pF at +30 mV (Figure 3D).

The curve of steady-state inactivation and recovery from inactivation of I_to_ were shown in Figures 3E,F. To elucidate the steady-state inactivation curve for I_to_, a two-pulse protocol was applied (panel A of Supplementary Figure 1). The experimental current traces were shown in panel B of Supplementary Figure 1. The simulated current traces from our model shown in panel C of Supplementary Figure 1 were more similar to the experiment traces compared with the current traces of the Hodgkin and Huxley (HH) formalization from the parent model (panel D of Supplementary Figure 1), implying a better description of our Markov chain model on the fast inactivation of I_to_. The steady-state inactivation curve and the recovery curve from inactivation produced from our model were all similar with experimental findings (Figures 3E,F). A temperature adjustment factor (Q_10_) used to rescale the model to body temperature was determined separately for channel conductance and kinetics. The Q_10_ of the channel kinetics were first determined as 1.8 from literature (Courtemanche et al., 1998; Brouillette et al., 2004), and then the Q_10_ factor for channel conductance was adjusted and set to 1.1 to match the current density at 32°C, which is ∼1.34 times of that at 22°C (Brouillette et al., 2004).

#### I_Kur_

I_Kur_ encoded by K_v1.5_, was identified in atrial myocytes from several species (Lomax et al., 2003a; Wettwer et al., 2004; Pandit et al., 2011). The kinetics of rapid activation and slow inactivation were reflected in the early current nomenclature, such as rapidly activating slowly inactivating current I_K,s_ (Bou-Abboud et al., 2000), or ultra-rapid K^+^ current I_Kur_ (Trépanier-Boulay et al., 2004), which is the currently accepted name for this current.

**FIGURE 4:**
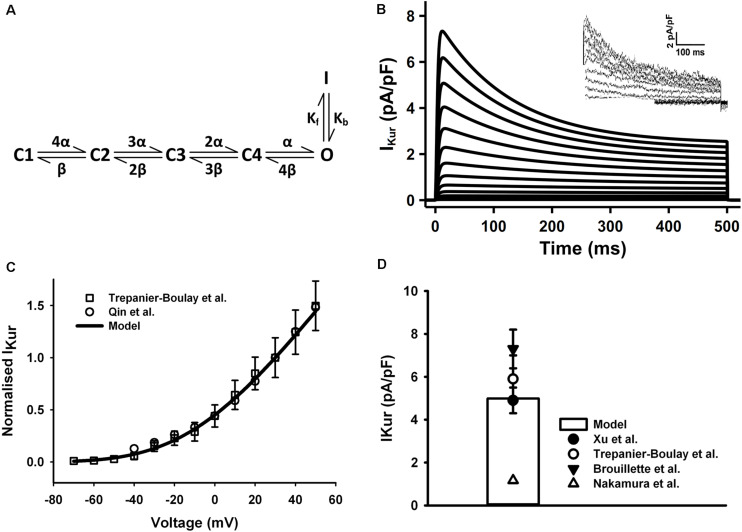
I_Kur_ in the atrial myocyte model and comparison with experimental data. **(A)** Schematic diagram of six-state Markov chain model of I_Kur_ (Zhou et al., 2012). **(B)** Current traces for the corresponding voltage clamp protocol. The inset shows the experimental record from Trepanier-Boulay et al. **(C)** I–V relationship, current densities were normalized to the current at +30 mV. **(D)** Current density when clamped to +30 mV (Experimental data: Xu et al., 1999c; Brouillette et al., 2004; Trépanier-Boulay et al., 2004; Nakamura et al., 2010; Qin et al., 2012).

The HH formulations of I_Kur_ for the mouse ventricular myocyte were replaced by a six-state Markov chain model from Zhou et al. (2012) (Figure 4A), in which the transition rates α and β were decreased to 1/8 of their original values in order to slow down the activation process of the I_Kur_ model to match to experimental data as shown in the Figure 4. The simulated current traces, normalized I–V curve, and peak current density of 5.1 pA/pF at +30 mV were similar to those seen in experiments (Figures 4B–D) (the current traces was simulated by using a protocol of 500-ms voltage steps in 10-mV increments between −70 and +50 mV from a holding potential of −80 mV). Since all experimental data were obtained under room temperature, the transition rates in the model were fitted to these data then adjust to the physiological temperature. Using the same method as for I_to_, the Q_10_ factor of activation and inactivation transition rates of I_Kur_ was set to 1.9 according to literature (Brouillette et al., 2004). Then the Q_10_ factor of channel conductance was chosen as 1.37 to fit the increase in current density at higher temperature (Brouillette et al., 2004; Nakamura et al., 2010), which is about 35 pA/pF at 22°C and 49 pA/pF at 32°C found in mouse ventricles.

#### I_Kr_

I_Kr_ encoded by ERG (KCNH2, K_v11.1_), has been proved to have a key role in the late repolarization of AP in several mammalian species (Sanguinetti and Jurkiewicz, 1990; Wang et al., 1994; Li et al., 1996). The presence and function of I_Kr_ in the mouse atrium has been demonstrated by Nakamura et al. (2010). Another study also shows that the relative mRNA level of ERG channel was higher and the amplitude of I_Kr_ was greater in mouse atria than in ventricles (Mewe et al., 2010).

**FIGURE 5:**
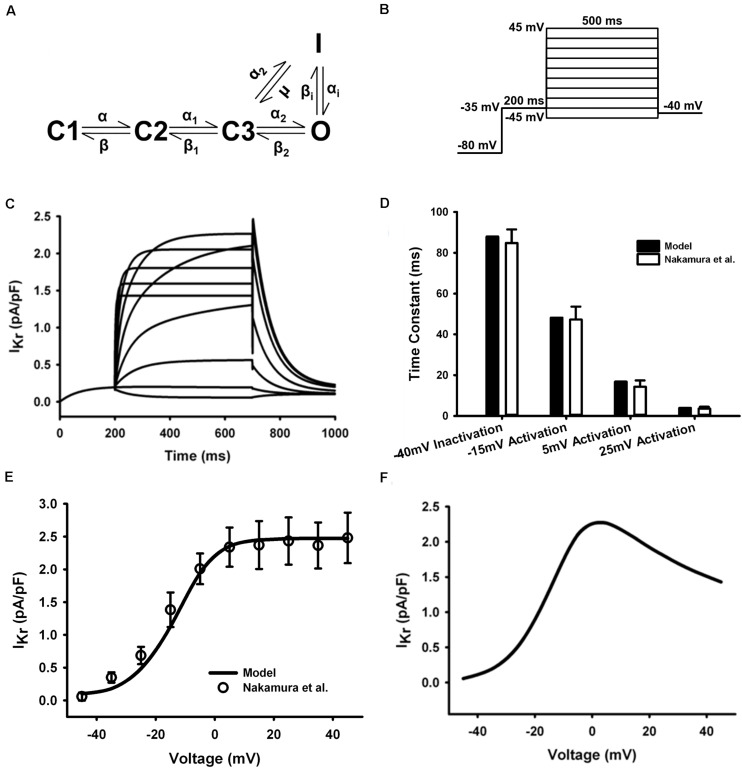
I_Kr_ in the atrial myocyte model and comparison with experimental data. **(A)** Five-state Markov chain model of I_Kr_. **(B)** The protocol used in the simulation. **(C)** Current traces for the corresponding voltage clamp protocol. **(D)** Time constants for inactivation and activation. **(E)** Tail I–V relationship. **(F)** Steady-state I–V relationship (Experimental data: Nakamura et al., 2010).

The HH formulations of I_Kr_ in the mouse ventricular myocyte were replaced with the five-state Markov chain model of Clancy and Rudy (2001) (Figure 5A) with modifications on transition rates to fit experimental measures. The model was stimulated with a voltage-clamp protocol shown in Figure 5B and resultant current traces are in Figure 5C. The time constants for inactivation and activation at several test potentials (Figure 5D) and the resultant tail current I–V curve (Figure 5E) were in agreement with experimental data (Nakamura et al., 2010) (the tail current were measured when the clamp voltage returning to −40 mV). The simulated steady-state I–V curve were also shown in Figure 5F. The model was fitted to experiment data at 37°C, so no temperature scaling factor was needed.

#### I_ss_

I_ss_ was found in the mouse ventricles and atria by Xu et al. (1999b,c). It is a 4-aminopyridine (4-AP) resistant current, which is distinctively different from I_to_ and I_Kur_ (Brouillette et al., 2004). The activation of I_ss_ is slow and it undergoes little or no inactivation (Xu et al., 1999a).

**FIGURE 6:**
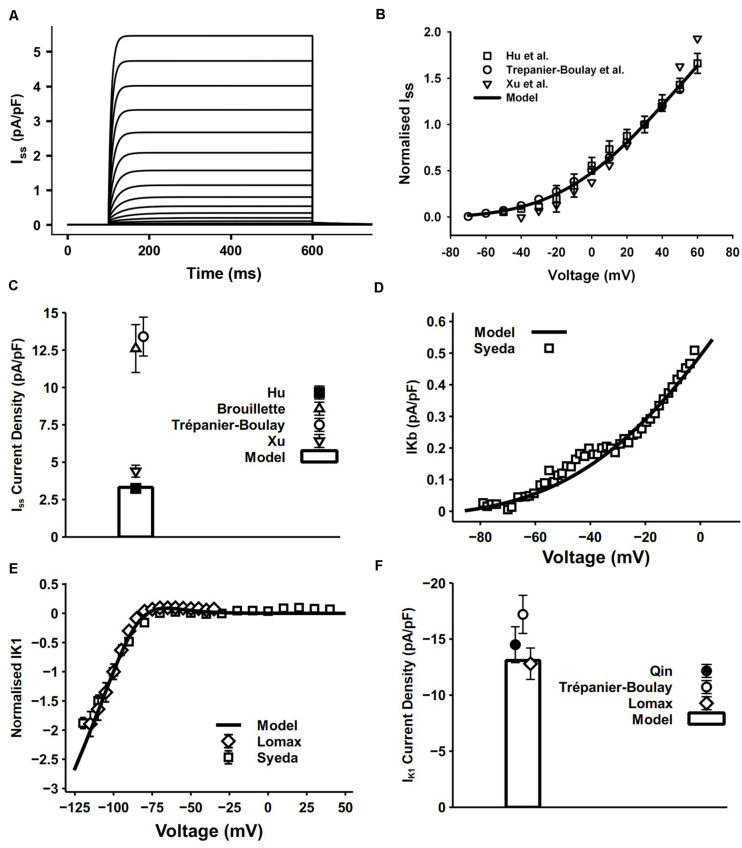
I_ss_, I_Kb_ and I_K1_ in the atrial myocyte model and comparison with experimental data. **(A)** Simulated current traces of I_ss_. **(B)** I–V relationship of I_ss_, values normalized to the current at +30 mV. **(C)** Current density of I_ss_ at +40 mV (experimental data: Xu et al., 1999c; Brouillette et al., 2004; Hu et al., 2005). **(D)** I–V relationship of I_Kb_. **(E)** I–V relationship for normalized I_K1_. **(F)** Current density of I_K1_ at –110 mV (experimental data: Lomax et al., 2003a; Trépanier-Boulay et al., 2004; Qin et al., 2012).

I_ss_ in our model was reformulated in the form of an HH formula to match its kinetics observed from atrial experimental measures. Since I_ss_ has no inactivating process, one activation gate x_ss_ is sufficient to reflect its relatively slow activation. The simulated current traces and normalized I–V relationship shown in Figures 6A,B were determined by using a protocol of 500-ms voltage steps in 10-mV increments between −80 and +60 mV from a holding potential of −80 mV. Considering the wide range of current density from experiments, conductance of I_ss_ was set to 0.049 nS/pF. The resultant peak current density 3.32 pA/pF at 30 mV and the comparison with experimental measurements are shown in Figure 6C. The simulated peak current density, normalized I–V curve and related current traces are similar to those seen in experiments. Experimental data were mostly acquired at 22°C, so a Q_10_ factor of 2.4 (Brouillette et al., 2004) was applied to the time constant of x_ss_ and channel conductance.

#### I_Kb_

In cardiac myocytes, the background potassium current I_Kb_ plays an important role in maintaining the polarized resting membrane potential (RMP) and intracellular K^+^ homeostasis. The recently discovered family of two pore domain K^+^ channels (K2Ps) have properties well suited to a role in mediating background K^+^ conductance (Gurney and Manoury, 2008). The model of I_Kb_ was described by a single Boltzmann equation fitted to experimental data measured by Syeda et al. (2016) at 37°C (Figure 6D). They applied 10 mM Ba^2+^ in isolated mouse myocytes and attributed the change in background current to twik-related acid-sensitive K^+^ (TASK) channels, which are in the K2P channel family. Since TASK channels display very fast activation and inactivation processes (Gurney and Manoury, 2008), no time constant was incorporated in our model.

#### I_K1_

The channel of I_K1_ encoded by Kir2.x stabilizes the resting potential and is responsible for shaping the initial depolarization and final repolarization of the action potential.

The I_K1_ equation in our model was based on that from DiFrancesco and Noble (1985) with model parameters adapted to mouse experimental data. The 500-ms pulse in the voltage-clamp protocol was from the holding potential of −80 mV to voltages from −150 to −40 mV. Currents were normalized to the magnitude at −100 mV. Simulated results were similar to the experimental data (Figures 6E,F).

#### Other Currents

##### I_Na_

The I_Na_ accounts for the fast upstroke of APs. Due to the absence of experimental data, in mouse atria, the channel kinetics and conductance were unaltered from the mouse ventricular model of Morotti et al. (2014). The resultant maximal upstroke velocity (dV/dt_max_) and AP amplitude were in good agreement with experiments.

##### I_NaL_

The kinetics of I_NaL_ were identical to the formulation described in Morotti et al. (2014), due to the lack of experimental data for the mouse atrium. The conductance of I_NaL_ was increased by 68% from the mouse ventricular model to fit the peak current density observed in the experiment (Lemoine et al., 2011).

##### I_KACh_

Acetylcholine activates I_KACh_, an inward rectifying K^+^ current. The molecular composition of cardiac I_KACh_ channels was identified as a heterotetramer consisting of two Kir3.1 (GIRK1) and two Kir3.4 (GIRK4) channel subunits. In the heart, I_KACh_ channels are primarily expressed in the atrioventricular and sinoatrial node and in the atria (Voigt et al., 2014). The I_KACh_ in our model was reparameterized from a HH formulation in a rabbit sinoatrial node model (Zhang et al., 2002). The simulated I–V relationship was compared with mouse atrial experimental data (Supplementary Figure 4).

##### I_KCa_

The small conductance Ca^2+^-activated K^+^ current, I_KCa_, is shown to contribute to repolarization in mouse atrial myocytes (Hancock et al., 2015). Three subtypes of the small conductance Ca^2+^-activated channels (SK1, SK2, SK3) are the carrier of I_KCa_, which are all sensitive to a selective SK channel blocker apamin (Adelman et al., 2012). We adopted the model of I_KCa_ from another mouse atrial cell model (Asfaw et al., 2020). The channel trafficking effect stimulated by β-adrenergic signaling system was removed.

##### I_NCX_

NCX (Na^+^-Ca^2+^ exchanger) encoded by the NCX family (NCX1, NCX2 and NCX3), is responsible for maintaining steady intracellular Ca^2+^ balance. It extrudes Ca^2+^ from the cytosol along with the plasma membrane Ca^2+^ pump (PMCA). As a reversible transporter, it also mediates Ca^2+^ entry in parallel with LTCCs (Blaustein and Lederer, 1999). Due to the lack of experimental results on NCX electrophysiological data for the mouse atria, the formulations for the NCX were kept unchanged.

##### I_NaK_

The I_NaK_ is responsible for maintaining the Na^+^ and K^+^ gradients between the cytosol and extracellular medium, and plays an important role in the restoration of the resting potential, and the generation and propagation of APs (Glitsch, 2001).

The conductance of I_NaK_ was adjusted according to the different regional expression of I_NaK_ in ventricles and atria. Schmidt et al. (1990) found that there were 50% fewer I_NaK_ in the atria than in the ventricles or septa by measuring the number of ouabain binding sites in porcine and canine hearts. In experiments conducted by Wang et al. (1993), the I_NaK_ activity in guinea pig atria was only one third of that in ventricles. In human atrial myocytes, the I_NaK_ activity was nearly 50% lower than that in ventricular myocytes as well (Wang et al., 1996). To adapt this change and also keep the intracellular Na^+^ concentration in physiological range, the maximum rate for the I_NaK_ was decreased by 40% in our model.

### Modeling of Intracellular Ca^2+^ Handling

The intracellular Ca^2+^ handling in this model is based on the framework developed by Shannon et al. (2004) and adopted by Morotti et al. into their ventricular model. This framework was utilized with some modifications.

Similar to the model of Shannon et al., the cell volume in our model was divided into four compartments (Figure 1B): the SR, the dyadic cleft, the sub-sarcolemma area (SL) and the bulk cytosol. Compared with ventricular cells, atrial cells usually have smaller size, higher percentage of cytosolic compartment, and lower percentage volume of other compartments. Thus, dimensions and parameters for the related structure in our model were modified accordingly (see Supplementary Document 1).

#### RyR Ca^2+^ Release (J_rel_)

The formulations for ryanodine receptor (RyR) were based on the model of mouse ventricular myocyte (Morotti et al., 2014), where the half maximal effective concentration (EC_50_) of the Ca^2+^ concentration in SR ([Ca]_SR_) on RyR was reduced by 10% to match the fractional Ca^2+^ release data from experiments.

The RyR Markov chain model has four states (panel A of Supplementary Figure 2), with strong dependence of RyR gating upon dyadic Ca^2+^ concentration ([Ca]_cleft_). During a normal calcium induced calcium release (CICR), some RyR channels promptly open (O) and rapidly transit into the inactivated states (I) as Ca^2+^ diffuses out of the dyadic cleft (panel B of Supplementary Figure 2). These channels then become resting inactivated states (RI) and eventually return to the resting states (R) (panel C of Supplementary Figure 2). The rate of Ca^2+^ release from the SR was 5.2 mM/s with a Ca^2+^ leakage rate of 0.36 μM/s (panel D of Supplementary Figure 2).

#### SR Ca^2+^ Reuptake (J_up_)

The main function of sarco/endoplasmic reticulum Ca^2+^-ATPase (SERCA) is the uptake of Ca^2+^ into SR. A proper model of SERCA is important for sustained normal CaTs and is highly related to the frequency dependent acceleration of relaxation (FDAR) of the cell model. The SERCA in our mouse atrial model was based on the model of Morotti et al. (2014) with a few modifications.

There is study showing that the decay of intracellular CaT in mouse atrial cells is far slower than in ventricular cells (Shanmugam et al., 2011) at 0.5-Hz pacing. Furthermore, Picht et al. (2007) suggested in their experiment in mouse ventricles that the Ca^2+^ removal rate via SERCA was greatly elevated at 4 Hz (2.1 times of that at 0.5 Hz) due to increased maximum turnover rate of SERCA (*V*_max_) without change in Ca^2+^ affinity of SERCA (K_m_). In our model, the *V*_max_ was reduced by 50% and a coefficient *K*_SERCA_ describing the effect of CaMKII at high pacing frequencies was adopted which is calculated by:

(1) $$K_{\text{SERCA}}=\frac{1}{{\left(0.0167/{\text{Ph}}_{\text{PLB}}\right)}^{12}}+1$$

where Ph_PLB_ denotes the level of phosphorylation of phospholamban (PLB) in percentage. And the forward mode Ca^2+^ affinity (K_mf_) was increased by 13%.

### Heterogeneity of Left and Right Atria

**FIGURE 7:**
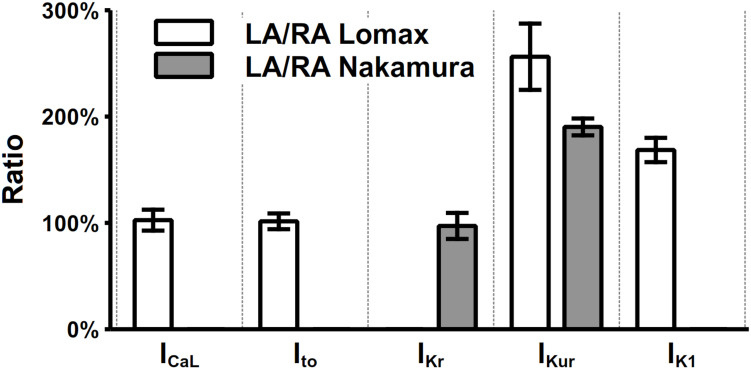
Heterogeneity of current densities between left and right atria. The current densities in left atria were normalized to their counterparts in the right atrium. The values of I_CaL_, I_to_, I_Kr_, I_Kur_, and I_K1_ were extracted at clamp voltages of 0, 35, 25, 35, and –105 mV, respectively.

Electrophysiological heterogeneity between mouse left and right atria has been investigated in many experimental works (Lomax et al., 2003a, b; Nygren et al., 2004; Verheule et al., 2004; Nakamura et al., 2010). Specifically, the current density differences between left and right atria of I_CaL_, I_to_, I_Kr_, I_Kur_, and I_K1_ were measured by Lomax et al. (2003a) and Nakamura et al. (2010), which are listed in Figure 7. The most prominent dissimilarity between left and right atrial cells lies in the current densities of I_Kur_ and I_K1_. There are no significant differences in the recovery from inactivation of I_to_ (Lomax et al., 2003a) and the time constant of activation of I_Kr_ (Nakamura et al., 2010). Considering these experimental findings, a model describing the mouse left atrial AP was derived from the constructed right atrial cell model by increasing the channel conductances of I_Kur_ and I_K1_ by 110 and 70%, respectively. Validation of the model is shown in the “Result” section.

Unless stated otherwise, simulations in this paper were based on the right atrial model.

### Validation of the Model

The developed model was validated by comparing the characteristics of simulated action potentials with those of experimental data obtained from mouse atrial cells in several perspectives, including the AP characteristics, CaT characteristics (with their rate dependences). Simulation results on the role of K^+^ currents in the AP were also compared to experimental data for validation or calibration purposes. The rate dependence of intracellular ion concentrations are also shown although cannot be treated as true validation, as the I_NaK_ was calibrated to reproduce physiological [K^+^]_i_ and [Na^+^]_i_. Because a large number of experimental data are referred in the “Result” section for validation, in order to avoid confusion, only experimental data explicitly from the mouse atrial cells will be presented in simulation figures for comparison purposes, while the experimental data from other species or other parts of the heart will only be referred in the text.

## Results

### Simulated AP and Currents

**FIGURE 8:**
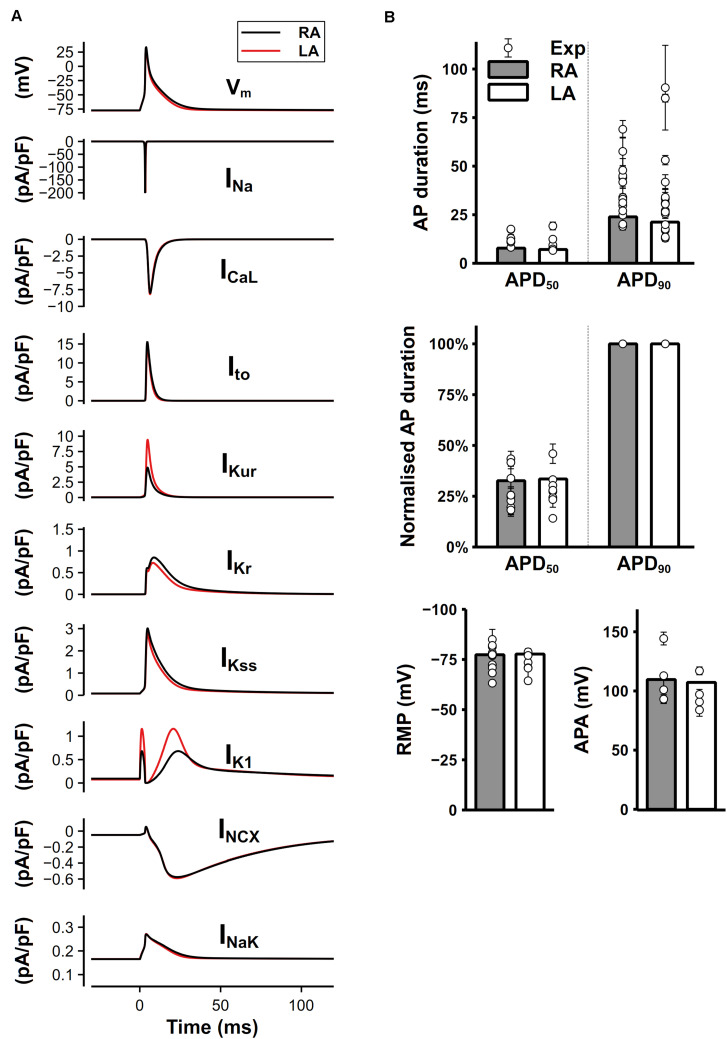
Simulated mouse atrial APs, currents and AP characteristics. **(A)** Mouse left and right atrial APs (shown as the membrane potential (V_m_) against time) and major underlying currents simulated at steady state with 1-Hz pacing. **(B)** Comparison between simulated (bars) and experimental records (circles) of AP characteristics.

The simulated time course of the mouse atrial AP and major underlying currents are shown in Figure 8A. The AP of left atrial cell model was slightly shorter than that of right atrial cell model due to the significantly greater I_Kur_ and I_K1_, while other potassium currents were smaller during the repolarization, producing a partial compensation to the large I_Kur_ and I_K1_. Figure 8B shows characteristics of simulated AP under 1-Hz pacing frequency, compared with experimental data. The dV/dt_max_ is about 210 V/s in the atrial model (not shown), which is similar to experimental results (Bao et al., 2016; Syeda et al., 2016). Experimentally measured APDs at 50 and 90% repolarization (APD_50_ and APD_90_, respectively) distributed in a wide range, which may be due to different experimental environments, such as temperature, pacing frequency, genetic backgrounds or strains. The computed APD_50_ by our model, though was marginally short, matched to the low bound of the experimental data range. The normalized APD based on the ratio of APD_50_ over APD_90_ were measured and presented (middle panel of Figure 8B), showing that the normalized APD_50_ from experiments and our model are in good agreement. Experimental data shown in Figure 8B are listed in Supplementary Table 1.

### Intracellular Ca^2+^ Handling

**FIGURE 9:**
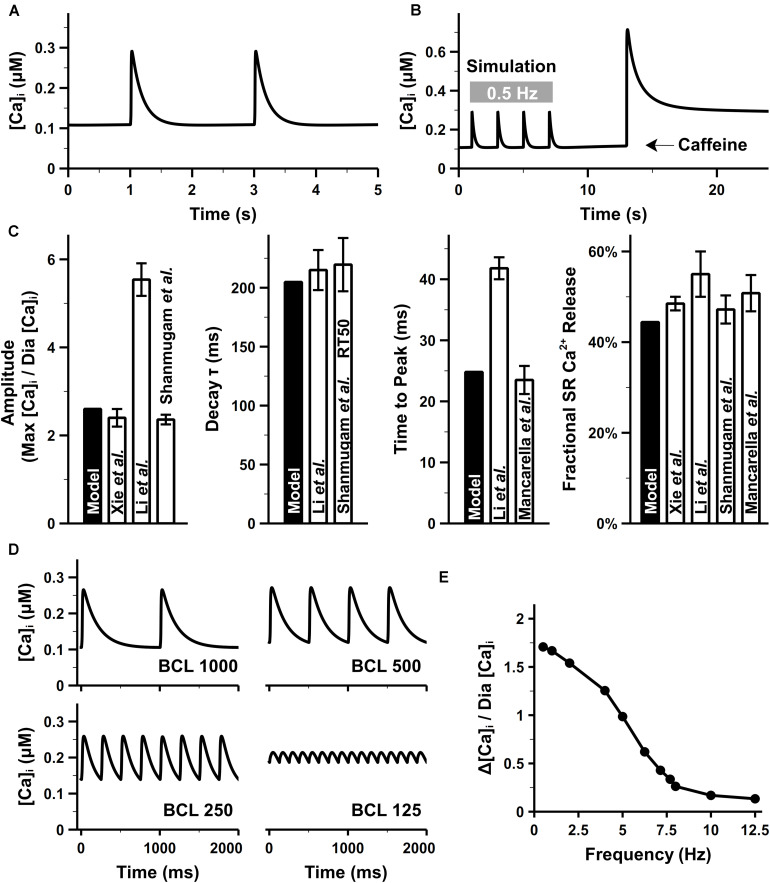
Atrial intracellular Ca^2+^ transient from simulation and experiments. **(A)** CaT under the pacing rate of 0.5 Hz. **(B)** Simulated caffeine-induced CaTs. **(C)** [Ca]_i_ amplitude, time to the peak of [Ca]_i_, time constant for the decay phase of [Ca]_i_, and fractional Ca^2+^ release, calculated at 0.5 Hz. **(D)** Time course of CaTs under 1-, 2-, and 4-, and 8-Hz pacing rate. **(E)** Amplitude of CaTs at various pacing rates. The diastolic Ca^2+^ concentration (Dia [Ca]_i_) was fixed as the value when the cell was paced at 0.5 Hz [experimental data: Xie et al. (2012) at body temperature, Li et al. (2005); Mancarella et al. (2008), Escobar et al. (2004); Walden et al. (2009), and Shanmugam et al. (2011)].

Our model well reproduced intracellular CaTs in mouse atrial cells. The CaTs under steady stimuli of 0.5 Hz were depicted in Figure 9A. When paced at 0.5 Hz, the diastolic Ca^2+^ concentration in the bulk cytosol ([Ca]_i_) was about 0.11 μM in our model, which was consistent with ventricular experimental findings (Glukhov et al., 2015). As suggested by Walden et al. (2009), there was no apparent difference in the diastolic [Ca]_i_ between murine ventricular and atrial myocytes. The caffeine-induced Ca^2+^ transient (Figure 9B) was similar with experiments by Xie et al. (2012). The effect of caffeine was simulated by increasing the opening probability of RyRs by 7.5 times and complete block of SERCA and I_Cab_ [the same as configurations in the parent mouse ventricular model (Morotti et al., 2014)]. Characteristics of CaTs were calculated from Figures 9A,B and shown in Figure 9C. The peak of amplitude of CaTs were ∼2.6 times higher than the basal level, which is comparable with various experimental data (Li et al., 2005; Shanmugam et al., 2011; Xie et al., 2012). The time constant for the decay phase and time to peak of CaT were also similar with the values reported in Li et al. (2005); Mancarella et al. (2008), and Shanmugam et al. (2011). The fractional SR Ca^2+^ release was calculated as the fraction of the maximum [Ca]_i_ before and after application of caffeine.

Figure 9D shows the simulation results of CaTs at various pacing frequencies. Although no direct atrial data available to compare, the amplitude of CaT (Figure 9E) decreased along with the increasing pacing frequency as seen in ventricular experiments (Picht et al., 2007). When the pacing frequency was between 1 and 3 Hz, there was no obvious change in the maximum [Ca]_i_, the reduced amplitude of CaT was due to the elevated diastolic [Ca]_i_. From 3 to 8 Hz, the total SR Ca^2+^ release was larger than the Ca^2+^ uptake by SERCA, leading to lower [Ca]_SR_ which in turn reduced the amplitude of every Ca^2+^ release until new steady states were reached. The new states had a markedly decreased maximum [Ca]_i_ and increased diastolic [Ca]_i_, therefore, a frequency-dependent attenuation of CaTs.

Ca^2+^ fluxes play a key role in the regulation of ECC (Bers, 2000; Eisner et al., 2000). The mouse atrial model can closely resemble the experimental data of Ca^2+^ fluxes. Supplementary Figure 2 shows the behavior of RyRs during a cardiac cycle. The peak value of J_rel_ was 95 mmol/(L SR)/s or 2.12 mmol/(L cytosol)/s, which was similar to experimental observation (Lukyanenko et al., 1998). The Ca^2+^ influx through LTCC peaked at 0.21 mmol/(L cytosol)/s, which was close to the experimental estimates of 0.30 mM/s (Bers, 2001). The Ca^2+^ fluxes of SERCA, NCX, and sarcolemmal Ca^2+^ pump are shown in Supplementary Figure 3. In our atrial model, the predominant removal mediator, SERCA, contributed 91.4% of the overall Ca^2+^ removal, with NCX significantly dropping to 7.2%. These results were closely similar to the experimental observations in the rat atrium (SERCA and NCX contributed 92.6 and 6.13% of the total Ca^2+^ removal) (Walden et al., 2009).

### Rate Dependence of Intracellular Ion Concentrations

**FIGURE 10:**
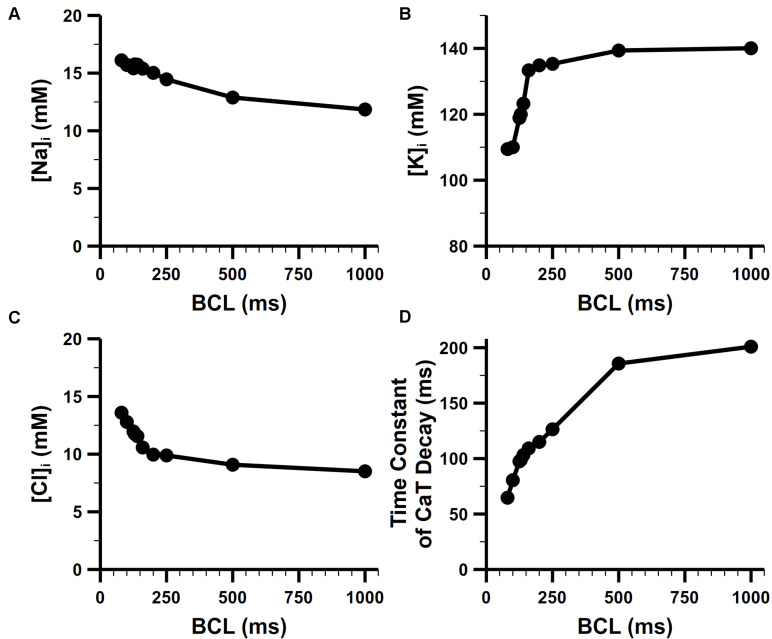
Steady-state rate dependence of intracellular ion concentrations. Rate dependence of **(A)** [Na]_i_, **(B)** [K]_i_, and **(C)** [Cl]_i_. **(D)** Rate dependence of the time constant of CaT decay.

The rate dependence in concentrations of intracellular Na^+^, K^+^ is shown Figures 10A,B. For the Na^+^ concentration in bulk cytosol ([Na]_i_), although no experimental data for mouse atria, our simulated results well fitted to the values found in murine ventricular myocytes. Without pacing, [Na]_i_ of the model stabilized at about 10.8 mM, which is quite similar to that of 10 mM in the rat ventricle (Despa et al., 2002). It has been found in experiments that [Na]_i_ increases along with the increasing pacing rates. In mouse ventricle, [Na]_i_ was 8.24 ± 4.9, 12.3 ± 4, and 15.1 ± 5.5 mM at 0.1, 0.5, and 3 Hz pacing rates, respectively (Wagner et al., 2006). In rat ventricle, [Na]_i_ was 11.2 ± 2.3 and 15 ± 1 mM at 0.5 and 2 Hz pacing rates, respectively. The [Na]_i_ of our model at various pacing frequencies were well distributed in the physiological range of 10–17 mM. The K^+^ concentration in the bulk cytosol ([K]_i_) of our model was about 140 mM at resting, and dropped to around 110 mM when paced at 10 Hz. This change in [K]_i_ significantly influence the equilibrium potential of K^+^ and then the RMP (details in the following section). These results implied that I_NaK_ magnitude was properly implemented, since I_NaK_ is responsible for pumping in K^+^ and Na^+^ out thus keeping the transmembrane gradient of Na^+^ and K^+^ concentration.

The Cl^–^ concentration in the bulk cytosol ([Cl]_i_) is also dynamic in our model. It changed from 8 mM to 14 mM when the pacing rate was increased from 1 Hz to 10 Hz (Figure 10C). The change in [Cl]_i_ generated a higher equilibrium potential of Cl^–^ and played a role in modifying the behavior of Cl^–^ currents (i.e., Ca^2+^-activated Cl^–^ current and background Cl^–^ current in our model). At 1 Hz, there was only an influx of the Cl^–^ forming a transient outward current when the cell was activated. At higher pacing rates, equilibrium potential of Cl^–^ was elevated to about −50 mV, leading to a Cl^–^ efflux in the diastolic phase of AP which slightly depolarized the RMP.

Figure 10D shows the rate dependence of the time constant of CaT decay. The decay became more rapid with increasing frequency, which reflected FDAR. At high pacing rates, there was enough CaMKII activated to phosphorylate PLBs (see the section of CaMKII-mediated phosphorylation in Supplementary Document 1 for more details) and enhance SERCA (by a larger coefficient *K*_SERCA_ described in section “SR Ca^2+^ Reuptake (J_up_)”). This boosted Ca^2+^ uptake contributed to the faster drop of CaT.

### Rate Dependence of Mouse Atrial APs

**FIGURE 11:**
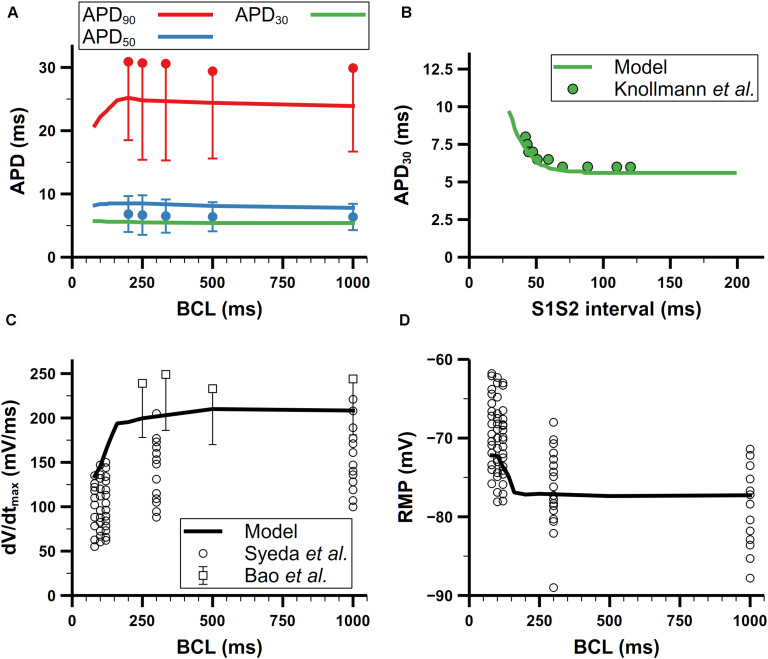
Rate dependence of mouse atrial APs. **(A)** Steady-state rate dependence of simulated APDs (lines) compared with experimental data (dots) from Bao et al. (2016). Red, blue, and green represent APD_90_, APD_50_, and APD_30_, respectively. **(B)** Simulated APD restitution curve under an S1S2 protocol compared with experimental data from Knollmann et al. (2007). The BCL of S1 was 130 ms. **(C)** Rate dependence of the maximum upstroke velocity. Data from Syeda et al. are all listed without using means and error bars. **(D)** Rate dependence of resting membrane potential (RMP) (All data from Bao et al. in panels **(A)** and **(C)** have symmetric errors, but some of them are shown with one-side error bars for clarity).

We further validated our mouse atrial model against experimental data of AP characteristics at different pacing frequencies (Figure 11). According to experimental data from Bao et al. (2016), the APD adaptation to pacing frequency of mouse atrial cells (shown in Figure 11A) is not distinct, which is quite similar with that in mouse ventricular cells (Wu and Anderson, 2002; Wagner et al., 2009). For restitution, we compared APD_30_ after S1 pacing at CL = 130 ms, followed by a single S2 extra-systolic stimulus delivered at various S1S2 intervals (Figure 11B). The APD_30_ restitution curve well reproduces the trend of increase when S1S2 intervals were being shortened. The steady-state rate dependence of mouse atrial dV/dt_max_ and RMP are shown in Figures 11C,D. At pacing frequencies lower than 4 Hz (CL > 250 ms), dV/dt_max_ and RMP remained almost stable; at higher pacing frequencies, dV/dt_max_ markedly decreased along with the increase of RMP, which is consistent with experimental data.

There are two likely causes for the elevated RMP at high pacing frequencies: slower late repolarization (defined as the phase of repolarization when membrane potential is below −60 mV) or depletion of [K]_i_. To investigate which one contributed most to the elevated RMP, we first clamped the [K]_i_ to 140 mM, which is the steady [K]_i_ under 1-Hz pacing, then stimulated the cell with 100-ms CL until the cell reached steady state. In this condition, the final RMP of the cell stayed below −78 mV, which means the rate dependence of the RMP disappeared. Moreover, this relatively low RMP also promoted the recovery of I_Na_ to such an extent that it eliminated the phenomenon of reduced I_Na_ at high pacing frequencies found in experiments by Syeda et al. (2016), and also led to more Na^+^ influx and [Na]_i_ overload. On the other hand, slower late repolarization may also raise the RMP due to not fully repolarized membrane potential when next stimulus comes. The late repolarization was slowed in our model because of reduced I_Kr_ and I_ss_ at high pacing rates. Below −60 mV, I_Kr_ and I_ss_ were reduced by about 50 and 40% at CL = 100 ms compared with those at CL = 500 ms, respectively. According to these changes in current amplitude, we tried to augment the I_Kr_ and I_ss_ to the level of slow pacing (CL = 500 ms) but the resultant RMP (−70 mV) was still elevated. Since it was the clamped [K]_i_ but not faster late repolarization that eliminated the rate dependence of RMP, our simulation suggested the [K]_i_ depletion was the predominant mechanism underlying the increase of RMP when the pacing rate was high.

### Rate Dependence of Ion Channel Currents

**FIGURE 12:**
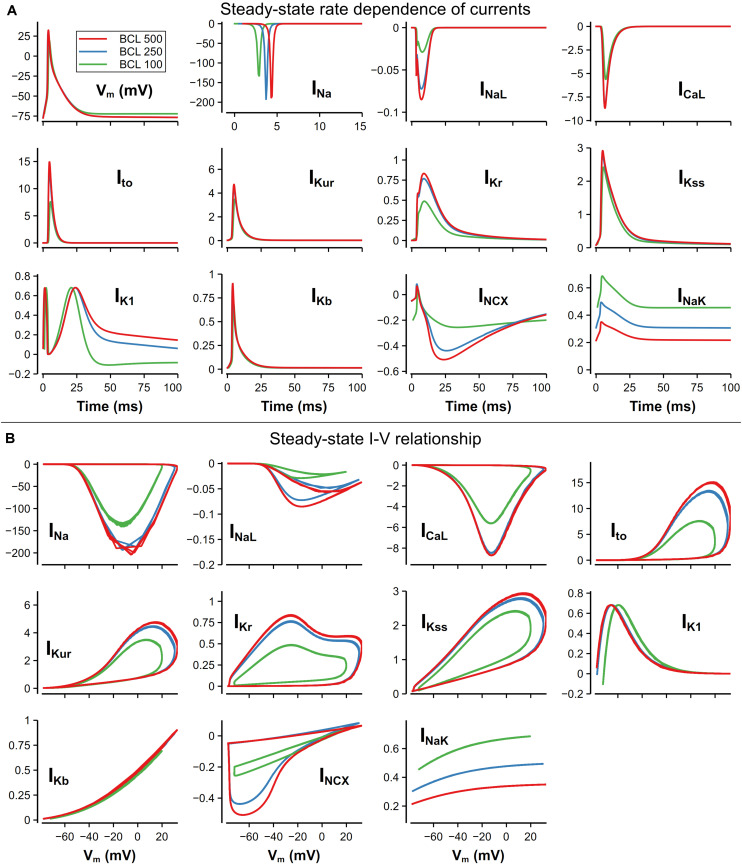
Steady-state rate dependence of currents and corresponding I–V relationship. The unit of all currents is pA/pF. **(A)** Currents against time at various CLs. The traces of I_Na_ were slightly moved along time to avoid overlapping. **(B)** I–V relationship of currents at various CLs.

For better explaining the mechanism underlying the rate dependence of AP, we investigated the role of each main channel current in the model. In Figures 12A,B, ion channel currents under 500, 250, and 100-ms CL were plotted against time or V_m_. These three CLs were chosen since the rate dependence of currents was unobvious when the CL was larger than 500 ms. There is still no obvious variation of the morphology of the AP with the CL from 500 to 250 ms, whereas distinct changes in I_K1_, I_NCX_, and I_NaK_ could be observed. Due to more APs evoked, much more Na^+^ entered the cell by I_Na_, resulting in the increased [Na]_i_ and then I_NaK_. On the other hand, the elevated [Na]_i_ suppressed the I_NCX_ and led to a rise of [Ca]_i_ (as in Figure 9D). I_K1_ was decreased because its role in the late repolarization was partly taken over by the larger I_NaK_.

With a further decrease in CL (from 250 to 100 ms), distinct changes on AP morphology could be observed. The RMP was elevated by about 5 mV, resulting from the decrease of [K]_i_. The overshoot (OS) decreased from 30 to 16 mV, which was mainly due to the decrease of I_Na_. I_Na_ decrease was also found by O’Hara et al. (2011) in their human ventricular cell model, but the reason of the decrease in our model was the elevated RMP leading to insufficient recovery of I_Na_ and limited Na channel availability, which was different to what was believed to be insufficient time for the I_N__a_ to recover. For other currents, because of the smaller OS, I_CaL_ and most currents for repolarization (I_to_, I_Kur_, I_Kr_) attenuated in varying degrees. It is also worth noting that the decrease in [K^+^]_i_ not only elevated the RMP, but also led to a reverse of I_K1_ due to the altered equilibrium potential of the potassium channel.

### Role of K^+^ Currents in the Genesis of Mouse Atrial APs

To further validate the model, the functional roles of main K^+^ currents were investigated by ion channel blocking simulations. Results are compared with experimental data.

#### Role of I_to_ and I_Kur_

I_to_ and I_Kur_ are both 4-AP-sensitive currents. Studies have demonstrated that relatively low concentrations (≤100 μM) of 4-AP predominantly blocks I_Kur_ while slightly affecting other types of K^+^ currents including I_to_ (Xu et al., 1999b). Since we cannot find experimental data for only I_to_ block, we consider the block of I_to_ and I_Kur_ by 4-AP in a combination manner.

**FIGURE 13:**
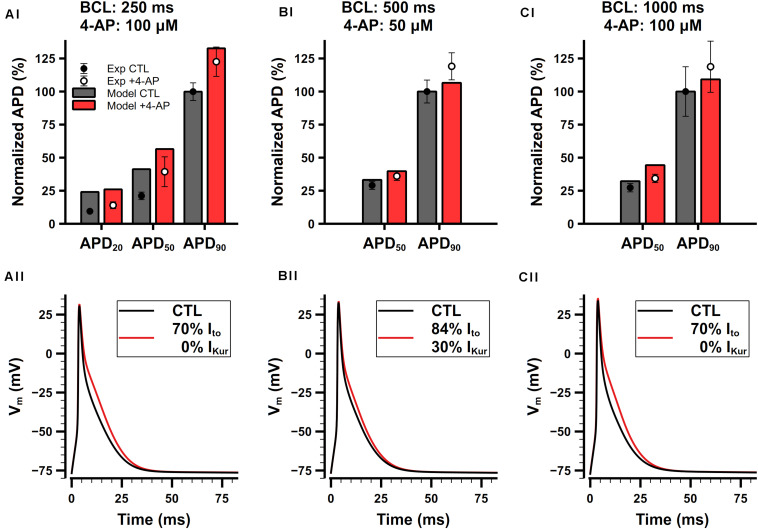
Role of I_to_ and I_Kur_ in the mouse atrial cell model. **(Ai,Aii)** Comparison of normalized APDs with experimental data **(Ai)** and simulated APs **(Aii)** before and after the application of 100-μM 4-AP. The cell was paced at 4 Hz. Experimental data was extracted from Trépanier-Boulay et al. (2004). **(Bi,Bii)** Comparison of normalized APDs with experimental data **(Bi)** and simulated APs **(Bii)** before and after the application of 50-μM 4-AP. The cell was paced at 2 Hz. Experimental data was extracted from Nakamura et al. (2010). **(Ci,Cii)** Comparison of normalized APDs with experimental data **(Ci)** and simulated APs **(Cii)** before and after the application of 100-μM 4-AP. The cell was paced at 1 Hz. Experimental data was extracted from Lomax et al. (2003a).

Studies have shown that 4-AP prolongs APs of mouse atrial cell (Lomax et al., 2003a; Nygren et al., 2004; Trépanier-Boulay et al., 2004; Nakamura et al., 2010). Experimental data from 3 different studies are shown in Figures 13Ai,Bi,Ci. Since different amount of 4-AP and pacing frequencies were used in these experiments, to make meaningful comparison with our model, it is necessary to first determine how much potassium currents were affected by different concentration of 4-AP. In experiments, Trépanier-Boulay et al. (2004) found that I_to_ was only blocked 5% by 100-μM 4-AP, whereas Nakamura et al. (2010) found that 100-μM 4-AP may affect I_to_ to a greater extent, so they chose to add 50-μM 4-AP to avoid its effect on I_to_. We also referred to the dose-dependent data of the effect of 4-AP on I_to_ and I_Kur_ in mouse ventricular cells (Xu et al., 1999b) and assumed that 4-AP should have similar effect in atrial cells. As a result, the percentages of I_to_ and I_Kur_ block were determined to: 16% I_to_ block and 70% I_Kur_ block under 50-μM 4-AP, or 30% I_to_ block and 100% I_Kur_ block under 100-μM 4-AP. The simulated APs and corresponding normalized APDs are shown in Figure 13. The block of I_to_ and I_Kur_ obviously prolongs APD_20_, APD_50_, and APD_90_. The simulation results were in good agreement with experimental data.

#### Role of I_Kr_

**FIGURE 14:**
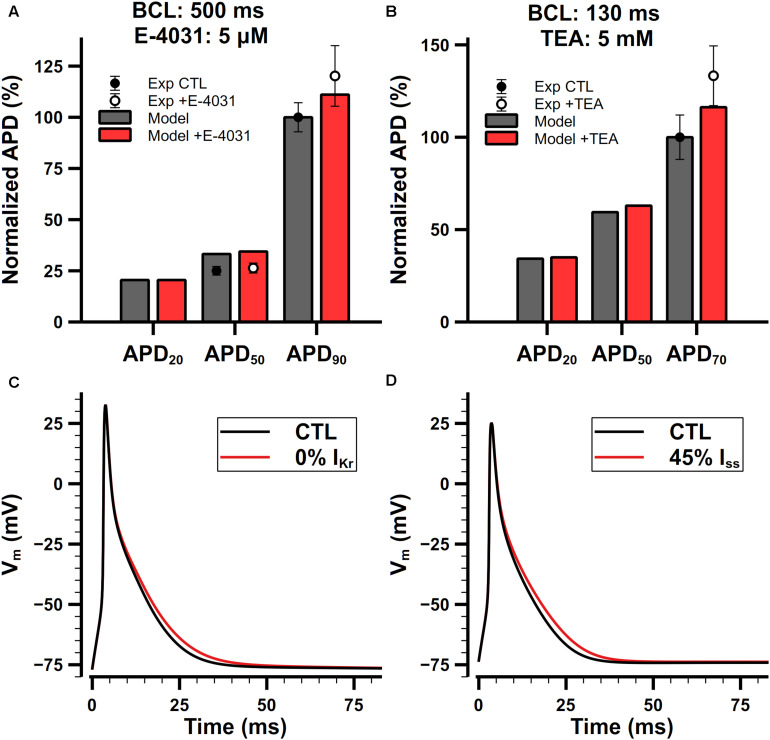
Role of I_Kr_ and I_ss_ in the mouse atrial cell model. **(Ai,Aii)** Comparison of normalized APDs with experimental data **(Ai)** and simulated APs **(Aii)** before and after the application of 5-μM E-4031. The cell was paced at 2 Hz. Experimental data was extracted from Nakamura et al. (2010). **(Bi,Bii)** Comparison of normalized APDs with experimental data **(Bi)** and simulated APs **(Bii)** before and after the application of 5-mM TEA. The CL of the pacing protocol was 130 ms. Experimental data was extracted from Nygren et al. (2004).

The role of I_Kr_ in the present model is to modulate the late phase of APs in mouse atrial cells. Normalized APDs and APs with or without application of E-4031 are shown in Figure 14A. I_Kr_ is extremely sensitive to E-4031 and can be fully blocked by only 5 μM of E-4031 (Nakamura et al., 2010). In our simulation, when paced at 2 Hz, the APD_20_, APD_50_, and APD_90_ of the cell model were 4.9, 8.1, and 24.4 ms under control condition, respectively. Full block of I_Kr_ didn’t change the APD_20_ but prolonged APD_50_ by 0.3 ms and APD_90_ by 2.6 ms. Exposure to E-4031 only affected the repolarizing of APs at levels lower than −25 mV, and it prolonged APD_90_ much more than APD_20_ and APD_50_. These results were in agreement with experimental findings (Nakamura et al., 2010).

#### Role of I_ss_

I_ss_ is not affected by 4-AP lower than 1 mM, but it is sensitive to tetraethylammonium (TEA). Nygren et al. (2004) examined the effect of 5-mM TEA on APDs in isolated mouse atrial preparations. Their results are shown in Figure 14Bi. Since we cannot find dose-dependent measurements in mouse atrial cells, we assume that the sensitivity of I_ss_ on TEA should be similar in the atrium and ventricle. It is reported that 25-mM TEA blocks 58% both I_ss_ and I_Kur_ in mouse ventricular cells (Xu et al., 1999a), but their dose dependences on TEA are quite different. Increasing the concentration of TEA to 125 mM will block I_Kur_ completely but only block 61% I_ss_ in mouse ventricular cells (Xu et al., 1999b), implying that the block of I_Kur_ by TEA has a steeper dose-dependent curve and lowering the dose of TEA should significantly decrease the extent of block on I_Kur_. We did a linear regression on the log scaled dose-dependent curve of the amplitude of I_ss_ and I_Kur_, and estimated that 5-mM TEA should block about 55% of I_ss_ and nearly no I_Kur_. Our simulation results were in good agreement with experimental data (Figures 14Bi,Bii).

## Discussion

### Novel Achievements

A new biophysically detailed model for the mouse atrial myocyte with considerations of electrical heterogeneity between the left and right atrial cells has been developed, which is the first model representing the spatially heterogeneous mouse atrial APs in the atria. Based on the modification on the mouse ventricular cell model developed by Morotti et al. (2014), the mouse atrial cell model presented here describes cellular Ca^2+^ and Na^+^ handling and their regulation by CaMKII and PKA. Three main potassium currents (I_to_, I_Kur_, and I_Kr_) were developed using Markov chain formalization and fitted to atrial experimental data, which allowed a better representation of atrial specific channel kinetics. Other membrane currents were also updated (I_Na_, I_NaL_, I_CaL_, I_ss_, I_K1_, I_KACh_, I_KCa_, I_PMCA_, I_NCX_, and I_NaK_) for atrial myocytes. All membrane currents were adjusted to physiological temperature either by fitting to data at 37°C or using temperature adjustment factors. Intracellular Ca^2+^ handling (RyRs, SERCA) and the CaMKII phosphorylation module were modified. Considering the heterogeneity between left and right atria, the atrial model was further extended to describe APs of mouse left atrial myocytes. The model was validated by its ability to reproduce the morphology and characteristics of APs and CaTs under various pacing frequencies, including APA, RP, APDs, etc. Characteristics of Ca^2+^ transients were comparable with those recorded from mouse atrial cells (see Figure 9). The role of main potassium currents in the genesis of the APs was investigated by simulation and validated with experimental data. The present model provides a basis for further simulating the conduction of excitation waves in the tissue of mouse atria in the future.

In the mouse atrial cell model constructed, we implemented Markov chain models instead of traditional HH models to model some of ion channels. Although the Markov chain model is more complex in the process of parameter fitting, and usually has more differential equations than the HH model and requires more simulation time, it helps to avoid limitations of the HH model in terms of model structure (Rudy and Silva, 2006). On the other hand, the Markov chain model is closely related to the conformation of ion channel proteins, which will be helpful in studying the change of channel function caused by drug action or gene defect in the future.

### Comparison With Other Models

The morphology of the simulated AP in our atrial myocyte was relatively short, similar to the report that mouse AP is different from that in most other species (e.g., dog, pig or human) (Kaese and Verheule, 2012). The main factor responsible for short mouse AP is the relative magnitude of the currents present. Mouse myocytes have large outward K^+^ currents, which ensure rapid repolarization. In our model, APD_90_ was shorter than that in the ventricular model (Morotti et al., 2014), which was in agreement with experimental observation (Knollmann et al., 2007). In most mammalian species, the atrial AP is characterized by a shorter early repolarization phase, resulting in a triangular wave shape, compared with the ventricular AP with its usually pronounced plateau phase (Grand et al., 1990). Our atrial model is able to reproduce this triangular atrial AP wave shape. Unlike other mammals, the atrial mouse AP was longer during the early repolarization phase (APD_25_), compared with that in the ventricle. Our model suggests that it is likely to be the result of reduced current density of I_to_ in atria.

Ca^2+^ handling in mouse myocardium has a very rapid mechanism (Bers, 2001). Unlike other mammals with longer APDs, the percentage of Ca^2+^ released from intracellular stores (mainly stored in SR) to cytosol during a Ca^2+^ transient is much higher in mouse myocytes, and the contribution of Ca^2+^ influx via LTCC smaller. Due to the relatively short APD of mouse myocytes, the Ca^2+^ removal during diastole is also very fast to ensure a sufficient refilling before the next contraction. Compared with the mouse ventricular cell model (Bondarenko et al., 2004; Morotti et al., 2014), the atrial model presented here has smaller I_CaL_ and I_NCX_, indicating that the normal ECC coupling requires less Ca^2+^ influx and thus less Ca^2+^ needs to be extruded from the cell. Therefore, CaTs in mouse atrial myocytes are more dependent on the intracellular Ca^2+^ store. Due to rapid SR Ca^2+^ release and reuptake, CaTs reach the peak and decay faster than in ventricular myocytes. This is in line with the observation in rat experiments that the time to peak of CaTs is earlier and the decay is faster in atria than in ventricles (Escobar et al., 2004). The difference between atrial and ventricular cells is also reflected in the contribution of three main proteins (SERCA, NCX, and PMCA) to Ca^2+^ removal. SERCA contributes more to calcium clearance in the atrial calcium transient, which is also consistent with experimental measurements (Walden et al., 2009).

Recently, another mouse atrial model was developed by Asfaw et al. (2020). Both our model and the Asfaw model provide a set of mathematical equations to simulate the action potential and CaT of mouse atrial myocytes. But there are some distinctive differences between the two as listed below. (1) Our model considers I_KACh_, and three main K^+^ currents (I_to_, I_Kur_, and I_Kr_), which are based on Markov chain formulations, which may be used as a tool for simulating functional impacts of gene mutations and drug interaction in future studies; (2) Our model also considers the electrical heterogeneity of left and right atrial myocytes (Figure 7), and thus extend the model to the left and right atrial cell model; (3) From the perspective of signal pathway, our model includes the effects of CaMKII on a variety of membrane currents, RyR, and SERCA, which plays an important role in the frequency dependent characteristics of the cell; (4) Though both models consider β-adrenergic receptor pathway, while the Asfaw model constructs β1- and β2-adrenergic receptors separately; and (5) In the subcellular structure, the Asfaw model considers caveolae and separates the SR into junctional SR and network SR, while our model does not. The simplification of our model on subcellular structure is described in more details in the limitation. From the perspective of model validation, our model was compared with more experimental data. Specifically, we collected the APDs of the left and right atrial myocytes, the characteristics of the CaT, the rate dependence of APD, RMP, dV/dt_max_, and also the data of the effects of K^+^ current block on the AP. In addition, the normal heart rate of mice is around 600 beats per minute (BPM) (Donner et al., 2011). Our model is stable under 12.5-Hz stimulation (or 750 BPM), while the Asfaw model was only tested with pacing rates up to 6 Hz.

Due to the limited kinetic data on the CaMKII phosphorylation in mouse atrial cells, the CaMKII module is mainly based on the previously published mouse ventricular model (Morotti et al., 2014). Experimental studies have shown that CaMKII can phosphorylate the α1C and the β2a subunits of the LTCC (Grueter et al., 2006). Our model is able to reproduce the quick CaMKII-dependent LTCC facilitation found in many experiments (Valverde et al., 2005; Huke and Bers, 2007). There are also experimental studies showing that CaMKII can phosphorylate the RyR and enhance its sensitivity to Ca^2+^ (Wehrens et al., 2004), and the SR Ca^2+^ leak and open probability of RyR increase after being phosphorylated by CaMKII (Chelu et al., 2009). Our model is able to mimic these effects by implementing a CaMKII-dependent factor of SR sensitivity and a factor on SR Ca^2+^ leak. PLB resides in the SR membrane and negatively regulates the activity of SERCA. PLB phosphorylation will release its inhibition on SERCA, allowing SERCA to more freely uptake Ca^2+^ from cytosol. Our simulation shows slow kinetics of PLB phosphorylation (see the section of CaMKII-mediated phosphorylation in Supplementary Document 1 for more details), which is observed experimentally (Huke and Bers, 2007). Compared with the ventricular model, the inhibitory effect of PLB on SERCA in our atrial model is relatively small, because the expression of PLB in murine atrial tissue is only half of that in ventricular tissue (Freestone et al., 2000).

### Intracellular Ionic Homeostasis

A valid cell model should be able to not only reproduce the action potential waveform but also ensure the ionic homeostasis at physiological heart rates. Under the prerequisite of keeping 4 types of ions dynamically changing (Na^+^, K^+^, Ca^2+^, Cl^–^), our model achieves ionic homeostasis under various pacing rates while all ion concentrations were in the physiological range. Dynamically changing ion concentrations enabled us to investigate the relationship between pacing frequency and cell behaviors. For example, we found in simulation that the [K]_i_ depletion at high pacing rates led to a series of influences which was reported by Syeda et al. (2016), including elevated RMP, decreased I_Na_, and reduced potassium currents. In all the main currents in our model, I_NaK_ and I_NCX_ play a key role of maintaining ionic homeostasis. I_NaK_ is the only current in charge of pumping Na^+^ out and K^+^ in, and I_NCX_ contributes 91% of the efflux of Ca^2+^ (the other part is extruded by PMCA). Along with increasing pacing frequency, more Na^+^ influx and K^+^ efflux lead to a higher [Na]_i_ hence larger I_NaK_ until new balance is constructed between the influx and efflux of Na^+^.

Because of the conservation law, when the steady state is achieved, the net fluxes of each type of ions should be 0 in a whole cycle of an AP. As adopted by other cell models, the stimulus current in our simulation is treated as a K^+^ current to avoid long-term K^+^ depletion. Without doing this, the final balanced [K]_i_ will decrease from 140 to 100 mM, leading to more than 5 mV elevation of the RMP, which is quite prominent. Our atrial cell model was carefully tuned to reproduce reasonable steady-state ion concentrations.

In simulations, a decrease of [K]_i_ was observed during fast pacing. To test whether the rundown of the intracellular K may occur in models as an artifact of the pacing stimulus, we further checked if any change in the stimulus waveform (i.e., different amplitudes of the stimulus current) would affect the intracellular K^+^ concentration. In the model, to evoke an action potential a pacing stimulus current as an analog of K^+^ current with a square wave form, similar to that used in the human ventricular cell model developed by O’Hara et al. (2011) was implemented. In the control case, the amplitude and duration of the stimulus current implemented were 10 pA/pF and 4 ms respectively, which was about 1.5 times of the excitation threshold of the model (at 7 pA/pF with 4-ms duration). In the test conditions, six different amplitudes of stimulus ranging from 80 to 200% of the original stimulus amplitude were implemented at the pacing rate of 10 Hz, and the steady state values of intracellular K concentration were measured. It was shown that up to 200% of the stimulus amplitude used in the study had no obvious effects on the computed [K]_i_ (<5%), suggesting that the rundown of [K]_i_ was not attributed to the artifact of stimulus.

### SERCA and FDAR

Frequency dependent acceleration of relaxation (FDAR) in murine hearts has been reported by many researchers (Kassiri et al., 2000; DeSantiago et al., 2002). This phenomenon is related to changes in myofilament properties (Chung et al., 2016) and in CaTs under high pacing rates. The changes of myofilament properties are beyond the aim of this paper, hereby we mainly discuss other possible roles contributing to frequency dependent acceleration of CaT decay (FDAD).

CaMKII plays an important role in the FDAD. The level of activated CaMKII is higher when the pacing is faster, and most targets phosphorylated by CaMKII directly influence the CaT. Among these targets, SERCA is predominant as it contributes more than 90% in the removal of [Ca]_i_, i.e., CaT decay, in murine cardiac myocytes (Walden et al., 2009). It is natural to guess that CaMKII amplifies SERCA under high pacing rates through some kind of mechanism. PLB may contribute to FDAD as it is a target phosphorylated by CaMKII and can regulate SERCA. There are reports showing that CaMKII phosphorylates PLB in a frequency-dependent manner (Hagemann et al., 2000; Valverde et al., 2005). But there are also studies suggesting temporal mismatch of the phosphorylation of PLB and the occurrence of FDAD (Valverde et al., 2005; Huke and Bers, 2007), which weakens the view that the frequency-dependent phosphorylation of PLB is the cause of FDAD. On the other hand, Picht et al. (2007) found that FDAD was caused by an increase in the *V*_max_ of SERCA with presence of CaMKII and unaltered K_m_ (Ca^2+^ affinity of SERCA), and was not related to the phosphorylation of PLB. From the perspective of modeling, if we want to guarantee the model has a 200-ms time constant of CaT decay under 0.5-Hz pacing, which is the value found in experiments (Li et al., 2005), the amplitude of SERCA has to be reduced to a degree that is too small at fast pacing rates to pump enough Ca^2+^ back in to the SR. In this case, the Ca^2+^ concentration in the cytosol and junctional area will be larger, leading to extra RyR Ca^2+^ releases during diastolic periods. Considering the exact mechanism of FDAD is still controversy, we chose to amplify the SERCA in an intuitive way that correlates the level of PLB phosphorylation with the *V*_max_ of SERCA as described in the “Model Development” section. It is proved by the simulation results that our model shows normal FDAD and CaTs.

### Limitations

#### Lack of Experimental Data

Although the mouse is a widely used animal model in the study of atrial arrhythmias, the quantification of the biophysical properties of mouse electrophysiology is still limited. In some instances, we need to use data obtained either at low temperature (lower than 36–37°C), from other regions of heart, or from different species, such as mouse ventricles, rats, canines, and rabbits. Most of the ion channels have to be fitted to experimental data acquired at non-physiological temperatures. Those experiments might be conducted at a temperature within the range of 20–37°C and by different groups. For I_ss_ and I_K1_, especially, only data at room temperature were available. The model of I_CaL_ was reformulated to fit with the mouse atrial I–V relationship, whereas the voltage-dependence of inactivation, the time constants of inactivation and recovery from inactivation were not validated due to the lack of available data. Also due to the lack of experimental data, the CaMKII and β-adrenergic modules were adapted from the parent mouse ventricular model. Another issue was the uncertainty about the accuracy of experimental data, which can vary by an order of magnitude, further limited the functionality of our model.

#### Limitation in the Cellular Structure

In ventricular myocytes, the well-developed transverse tubules (T-tubules) uniformly spread depolarization into the cell, resulting in a nearly synchronous SR Ca^2+^ release throughout the entire cell (Cheng et al., 1993). Atrial myocytes in small species, however, either lack a well-developed T-tubule network or have an irregular internal transverse-axial tubular system (Kirk et al., 2003) (Supplementary Figure S1B in Supplementary Document 1). As a result, the initial rise of the Ca^2+^ transient starts from the periphery of the cell and then the inner Ca^2+^ are released sequentially toward the cell center with decreasing amplitude (Trafford et al., 2013). Since the Ca^2+^ handling in our atrial model is based on the framework of ventricular myocytes (Shannon et al., 2004), SR Ca^2+^ releases only happen in the junctional area, and Ca^2+^ fluxes from the junctional area to SL and SL to cytosol are proportional with the Ca^2+^ gradients. This structure cannot mimic the Ca^2+^ sparks propagating in the cytosol, therefore underestimates the diffusion speed of Ca^2+^. Increasing the diffusion coefficient cannot be a solution because the Ca^2+^ sparks exist in only a short period, which cannot be mimicked by a larger but fixed diffusion coefficient through the whole AP.

#### Computational Cost

The Markov chain model as a widely used modeling paradigm, its advantages have been discussed extensively (Rudy and Silva, 2006; Fink and Noble, 2009). For example, Markov chain model can better mimic the kinetics of ion channels or proteins, and can be more easily extended to simulate the effects of drugs or gene mutations. However, Markov chain model is generally more complex than HH model, which consists of more differential equations and needs more computation. In the absence of extensive experimental data there are also some limitations in determining the model parameters for the complex system as Markov chain models, and the predictive capabilities of models fit only to experimental I–V curves are limited (Clerx et al., 2019; Whittaker et al., 2020).

Our first intention in constructing the presented model with Markov chain formulations for some ion channels was to describe the electrophysiological properties of mouse atrial myocytes as well as possible, and modulations to them by signaling pathways as detailed as possible, at a cost of non-optimized computational performance of the model. In fact, it costs about 0.8 s for 1 s simulation (pacing the model at 1 Hz) on a 2.8 GHz Core i7 CPU with one core. In multi-dimensional simulation, the computation can be paralleled to significantly reduce the time consumption. The computational time is nearly linear to the number of myocytes, which means that, it may take about 10 s for simulating 1 s electrical activity of a network of 100 myocytes with an 8-core CPU. Since modern CPUs are more and more powerful, we think the performance of our model is acceptable for most applications. If higher performance is needed, one could choose to replace some ion channels with HH models or remove the CaMKII or β-adrenergic pathways from the model.

### Conclusion

A new model accounting for the heterogeneous electrical action potentials between the left and the right atrial cells has been developed. The model was derived from, and validated against experimental data obtained from mouse atrial cells. The developed model provides a novel basis for further study of possible mechanisms underlying atrial fibrillations.

## Data Availability Statement

All datasets presented in this study are included in the article/Supplementary Material.

## Author Contributions

HZ conceived the study. WS, SZ, and HZ designed most of the study, performed the simulations and analyses, and wrote most of the manuscript. WW contributed to the design of simulations, figure design, and manuscript writing. WS aided SZ with reuse of codes from his previous study. HZ, WS, and KW supervised the project. All authors contributed to the article and approved the submitted version.

## Conflict of Interest

The authors declare that the research was conducted in the absence of any commercial or financial relationships that could be construed as a potential conflict of interest.
