# Supplementary material for: A Mathematical Model of the Mouse Atrial Myocyte With Inter-Atrial Electrophysiological Heterogeneity
Source: Front Physiol. 2020 Aug 6;11:972. doi: 10.3389/fphys.2020.00972 (PMC7425199; doi:10.3389/fphys.2020.00972)
Supplement: Supplementary file 1 [file Data_Sheet_1.docx]

Supplementary Document I: More Details of the Model Development

# Numerical Method and Details of Simulation

The original MATLAB code of Morotti’s ventricular model [1] was rewritten in the C programming language. The C code of unchanged modules from the ventricular model were validated and verified by their abilities to generate the same results at every time step as the MATLAB code. Nonlinear curve fittings in developing ion channel models were performed using a least-square method provided by the LMFIT package and implemented in Python. Ordinary differential equations were solved by Sundials CVODE package using a linear multistep method with a backward differentiation formula. Unless stated otherwise, APs were obtained by pacing the model at indicated frequencies at steady state when relative differences of intracellular ion concentrations between beats were lower than one hundred thousandth of the basal level. It took about 0.7 s for 1 s simulation on a 2.8 GHz Core i7 CPU. Normally, the model needs to be run for about 5 minutes (cell time) to get steady.

# Summarised Changes on the Parent model

## Cell Geometry

A mouse atrial myocyte is smaller than a ventricular myocyte. Compared with the ventricular myocyte, both the cell length and the cell width are smaller for the atrial myocyte. Based on experimental measurements [2–4], the cell length in our model was reduced from 100 μm in the ventricular myocyte to 90 μm, and the cell width was reduced from 21 μm to 13 μm (Fig S1B). Changes were listed in Table S1.

**Table S1. Cell geometry compared with the ventricular model of Morotti *et al.*** [1]**.** Parameters denoted with ‘†’ were derived from experimental sources.

|  | Ventricle | Atrium | Reference |
| --- | --- | --- | --- |
| Cell Length (µm) | 100 | 90 | [2] |
| Cell Radius (µm) | 10.25 | 6.5 | [5] |
| Cell Volume V_cell_ (pL) | 33 | 11.95 | [5]† |
| Cell Surface Area A_cell_ (µm^2^) | 20000 | 8290 | [5]† |
| Cell Capacitance C_mem_ (pF) | 200 | 50 | [6] |
| Cytosol Volume (V_myo_ / V_cell_) | 65% | 79% | [3] |
| SR Volume (V_sr_ / V_cell_) | 3.5% | 1.76% | [7] |
| SL Volume (V_SL_ / V_cell_) | 2% | 1.7 | [7] |
| Cleft Volume (V_junc_ / V_cell_) | 0.0539% | 0.01997% | [7] |


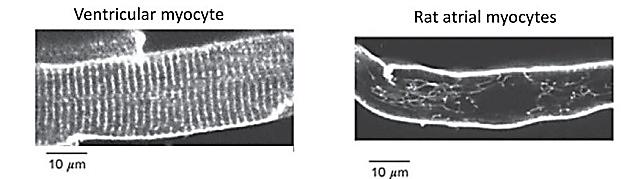

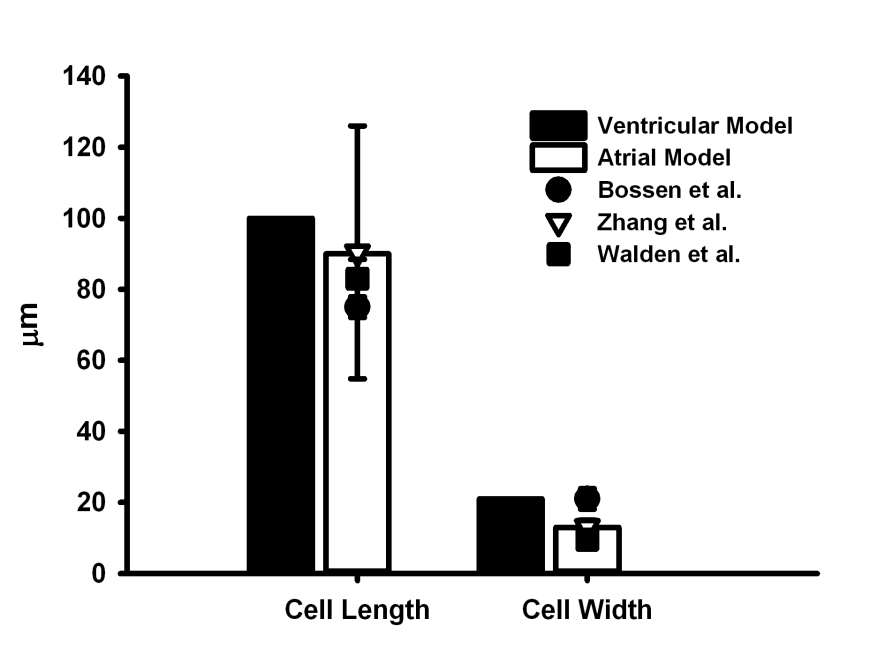


**A**

**B**

**Ventricular**

**Atrial**

**A**

**B**

**Figure S1. Cell geometry differences between ventricular and atrial myocytes.** (A) Membrane staining of rat ventricular and atrial myocytes showing a regular T-tube network in ventricular myocytes, and an atrial myocyte where T-tubes are absent. Figures are cited from [8]. (B) Cell dimension differences. The filled bar is the value used in the ventricular model of Morotti *et al.* [1]. The empty bar is the one used in our atrial model. Experimental measures for murine atrial cells are from Bossen *et al.* [3], Zhang *et al.* [2] and Walden *et al.* [4].

## Currents

**Table S2. Changes of parameters in currents.** Parameters denoted with ‘§’ were determined by estimation against data from cited sources.

| Target | Changes | Reference |
| --- | --- | --- |
| I_CaL_ | Current magnitude decreased to 1/4; steady-state; activation rate was modified to fit the experimental data; max phosphorylation of mode 2 channel was increased from 10% to 20% | [9,10] |
| I_to_ | Adapted Markov model (MM) and matched kinetics to the experimental data | [7,11] |
| I_Kur_ | Adapted MM and matched kinetics to the experimental data | [11,12] |
| I_Kr_ | Adapted MM and matched kinetics to the experimental data | [13,14] |
| I_Ks_ | Removed | [6] |
| I_K1_ | Matched kinetics to the experimental data | [7] |
| I_Kb_ | Added based on experimental data | [15] |
| I_KACh_ | Newly added |  |
| I_KCa_ | Newly added |  |
| I_Cap_ | Current magnitude reduced by 28% | [4]§ |
| I_NaK_ | Current magnitude reduced by 40% | [16] |

## Ca^2+^ Handling

**Table S3. Changes of parameters for Ca^2+^ handling.**

| Target | Changes | Reference |
| --- | --- | --- |
| J_rel_ | EC_50_ of [Ca]_SR_ on RyR was reduced by 10% |  |
| J_up_ | V_max_ reduced by 50%; phosphorylation of PLB was considered | [17] |

# CaMKII-mediated phosphorylation

CaMKII-mediated phosphorylation levels (percentage of total phosphorylated substrates) increase with higher pacing frequency, although levels and kinetics are quantitatively distinct. **Figure S2** shows the time course of CaMKII phosphorylation profiles during stimulation at low (1 Hz) and higher (4 Hz) frequency. When initially pacing at 1 Hz and switching to 4 Hz at 5 s in the atrial model, LTCC exhibited moderate (~60%) to high (88%) phosphorylation activity within a few beats, whereas RyR and PLB took more beats to achieve a steady level of phosphorylation. RyR phosphorylation level was increased from ~20% to 29% and the level in PLB was increased from ~0.6% to 1.6%.


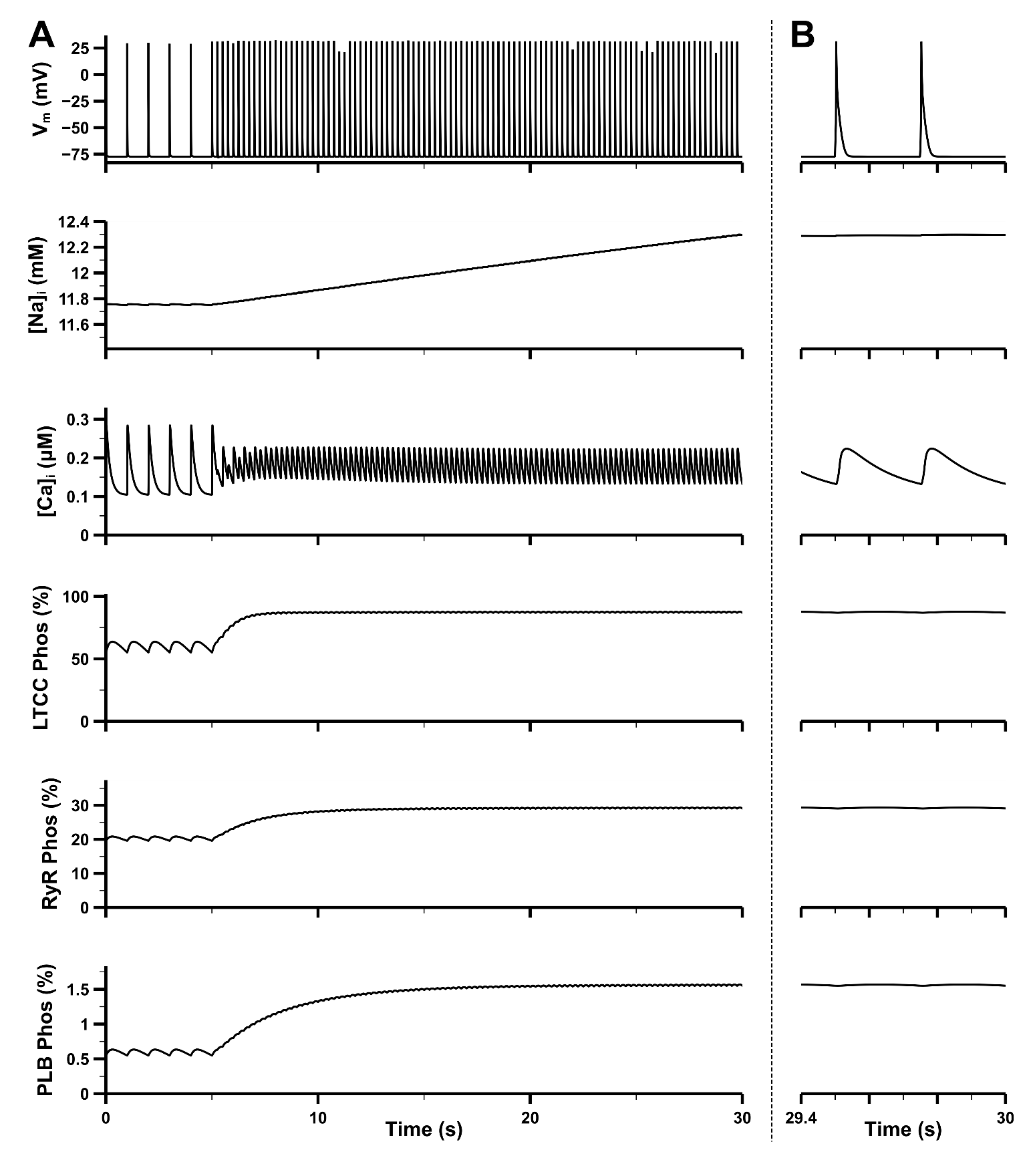


**Figure S2. Time course of CaMKII-mediated phosphorylation in the atrial cell model.** The model was paced to steady state at 1 Hz and switched to 4 Hz at 5 s. (A) Time courses of AP, [Na]_i_, [Ca]_i_, and the percentage of phosphorylated LTCC, RyR, PLB by CaMKII. (B) Stretched time courses from A. Last 2 beats are shown.

The frequency-dependent phosphorylation for LTCC, RyR and PLB are shown in **Figure S3**. There were no significant differences for the phosphorylation of LTCC and RyR between the two cell types. Phosphorylation of PLB in the atrial model, however, exhibited a higher level of phosphorylation as compared with the ventricular model: this is because a lesser amount of PLB was expressed in the atrial model. Although PLB showed a higher percentage of phosphorylation, its overall inhibitory effect on SERCA was actually weakened because of the reduced PLB, as compared with that in the ventricular myocyte model.


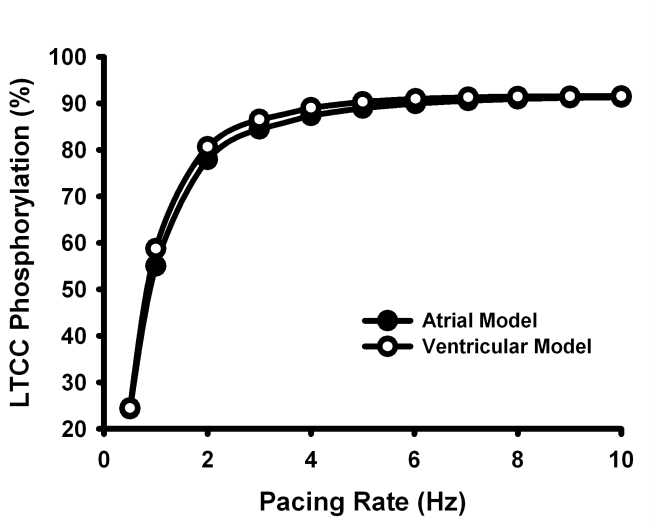

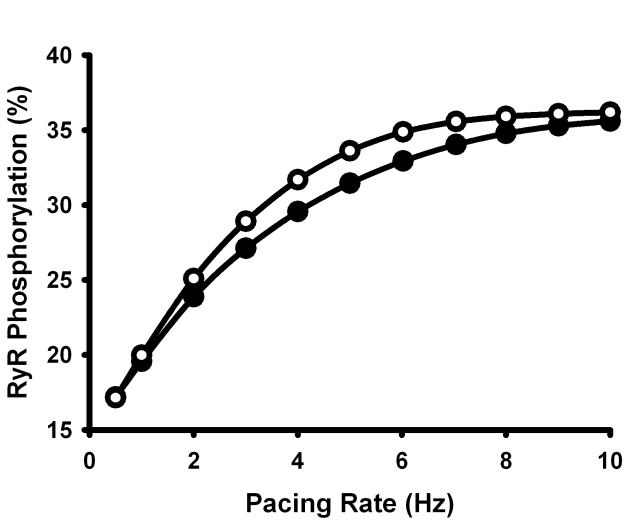

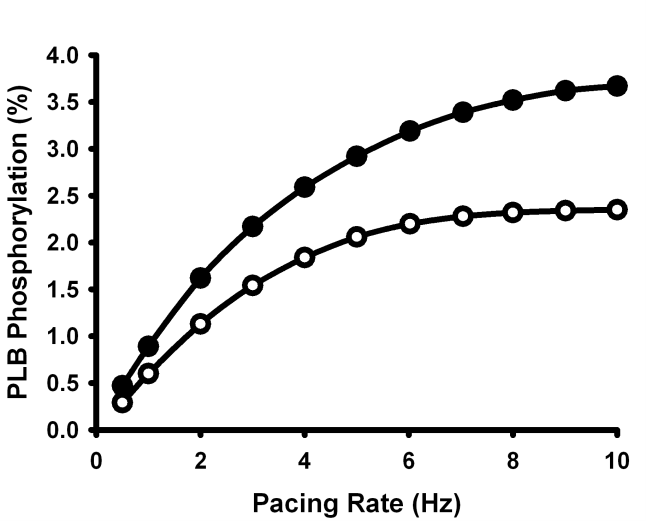


**B**

**A**

**C**

**Figure S3. Comparison of frequency-dependent CaMKII-mediated phosphorylation in the mouse atrial and the parent ventricular cell model.** Percentages of phosphorylated LTCC (A), RyR (B), and PLB (C) under different pacing rates.

# References

1. Morotti S, Edwards AG, McCulloch AD, Bers DM, Grandi E. A novel computational model of mouse myocyte electrophysiology to assess the synergy between Na^+^ loading and CaMKII. J Physiol. 2014;592: 1181–1197. doi:10.1113/jphysiol.2013.266676

2. Zhang Y, Schwiening C, Killeen MJ, Zhang Y, Ma A, Lei M, et al. Pharmacological changes in cellular Ca^2+^ homeostasis parallel initiation of atrial arrhythmogenesis in murine langendorff-perfused hearts. Clin Exp Pharmacol Physiol. 2009;36: 969–980. doi:10.1111/j.1440-1681.2009.05170.x

3. Bossen EH, Sommer JR, Waugh RA. Comparative stereology of mouse atria. Tissue Cell. 1981;13: 71–77. doi:10.1016/0040-8166(81)90039-2

4. Walden A, Dibb K, Trafford A. Differences in intracellular calcium homeostasis between atrial and ventricular myocytes. J Mol Cell Cardiol. 2009;46: 463–473.

5. King JH, Zhang Y, Lei M, Grace AA, Huang CL-H, Fraser JA. Atrial arrhythmia, triggering events and conduction abnormalities in isolated murine RyR2-P2328S hearts. Acta Physiol. 2013;207: 308–323. doi:10.1111/apha.12006

6. Mancarella S, Yue Y, Karnabi E, Qu Y, El-Sherif N, Boutjdir M. Impaired Ca^2+^ homeostasis is associated with atrial fibrillation in the α1D L-type Ca^2+^ channel KO mouse. Am J Physiol - Heart Circ Physiol. 2008;295: H2017–H2024. doi:10.1152/ajpheart.00537.2008

7. Lomax AE, Kondo CS, Giles WR. Comparison of time- and voltage-dependent K^+^ currents in myocytes from left and right atria of adult mice. Am J Physiol - Heart Circ Physiol. 2003;285: H1837–H1848. doi:10.1152/ajpheart.00386.2003

8. Trafford AW, Clarke JD, Richards MA, Eisner DA, Dibb KM. Calcium signalling microdomains and the t-tubular system in atrial mycoytes: potential roles in cardiac disease and arrhythmias. Cardiovasc Res. 2013;98: 192–203. doi:10.1093/cvr/cvt018

9. Xie L-H, Shanmugam M, Park JY, Zhao Z, Wen H, Tian B, et al. Ablation of sarcolipin results in atrial remodeling. Am J Physiol - Cell Physiol. 2012;302: C1762–C1771. doi:10.1152/ajpcell.00425.2011

10. Mahajan A, Shiferaw Y, Sato D, Baher A, Olcese R, Xie L-H, et al. A Rabbit Ventricular Action Potential Model Replicating Cardiac Dynamics at Rapid Heart Rates. Biophys J. 2008;94: 392–410. doi:10.1529/biophysj.106.98160

11. Zhou Q, Bett GCL, Rasmusson RL. Markov Models of Use-Dependence and Reverse Use-Dependence during the Mouse Cardiac Action Potential. PLOS ONE. 2012;7: e42295. doi:10.1371/journal.pone.0042295

12. Trépanier-Boulay V, Lupien M-A, St-Michel C, Fiset C. Postnatal development of atrial repolarization in the mouse. Cardiovasc Res. 2004;64: 84–93. doi:10.1016/j.cardiores.2004.06.002

13. Nakamura H, Ding W-G, Sanada M, Maeda K, Kawai H, Maegawa H, et al. Presence and functional role of the rapidly activating delayed rectifier K^+^ current in left and right atria of adult mice. Eur J Pharmacol. 2010;649: 14–22. doi:10.1016/j.ejphar.2010.08.025

14. Clancy CE, Rudy Y. Cellular consequences of HERG mutations in the long QT syndrome: precursors to sudden cardiac death. Cardiovasc Res. 2001;50: 301–313. doi:10.1016/s0008-6363(00)00293-5

15. Syeda F, Holmes AP, Yu TY, Tull S, Kuhlmann SM, Pavlovic D, et al. PITX2 Modulates Atrial Membrane Potential and the Antiarrhythmic Effects of Sodium-Channel Blockers. J Am Coll Cardiol. 2016;68: 1881–1894. doi:10.1016/j.jacc.2016.07.766

16. Wang J, Schwinger R, Frank K, Müller-Ehmsen J, Martin-Vasallo P, Pressley TA, et al. Regional expression of sodium pump subunits isoforms and Na^+^-Ca^++^ exchanger in the human heart. J Clin Invest. 1996;98: 1650.

17. Picht E, DeSantiago J, Huke S, Kaetzel MA, Dedman JR, Bers DM. CaMKII inhibition targeted to the sarcoplasmic reticulum inhibits frequency dependent acceleration of relaxation and Ca^2+^ current facilitation. J Mol Cell Cardiol. 2007;42: 196–205. doi:10.1016/j.yjmcc.2006.09.007
