# Supplementary material for: A Mathematical Model of the Mouse Atrial Myocyte With Inter-Atrial Electrophysiological Heterogeneity
Source: Front Physiol. 2020 Aug 6;11:972. doi: 10.3389/fphys.2020.00972 (PMC7425199; doi:10.3389/fphys.2020.00972)
Supplement: Supplementary file 2 [file Data_Sheet_2.docx]

Supplementary Document II: Model Equations

# Parameters





## Cell geometry





## Fractional currents

Fractions of a current in the SL or junction.





Surface area of SL; Number of junctions; Surface area of all junctions.





## Constant concentrations

|  | Extracellular Na^+^ concentration [mM] |
| --- | --- |
|  | Extracellular K^+^ concentration [mM] |
|  | Extracellular Ca^2+^ concentration [mM] |
|  | Extracellular Cl^-^ concentration [mM] |
|  | Intracellular Cl^-^ concentration [mM] |
|  | Intracellular Mg^2+^ concentration [mM] |

## Reversal potential





# Phosphorylation module

The description of Ca^2+^/calmodulin-dependent activation of CaMKII and the β-adrenergic signaling pathway were left unaltered from the parent model [1–3]. Parameters of phosphorylation are accessed by the other part of the model.

|  | Phosphorylation of LTCC by CaMKII |
| --- | --- |
|  | Phosphorylation of RyR by CaMKII |
|  | Phosphorylation of PLB by CaMKII |
|  | Phosphorylation of LTCC alpha subunit by PKA |
|  | Phosphorylation of LTCC beta subunit by PKA |
|  | Phosphorylation of PLB by PKA |
|  | Phosphorylation of RyR by PKA |
|  | Phosphorylation of TnI by PKA |
|  | Phosphorylation of Kur by PKA |
|  | Phosphorylation of PLM by PKA |

# Currents

## Stimulus

The amplitude of stimulus is set to -10 pA/pF and the duration is 4 ms. The carrier of the stimulus is K^+^.

## I_Na_




















## I_NaL_











## I_CaL_

Four different Markov chain models were run for I_CaL_ because the LTCC was divided into four groups: 1) mode 1 LTCC at SL; 2) mode 2 LTCC at SL; 3) mode 1 LTCC at junctional area; 4) mode 2 LTCC at junctional area. Each of them has 6 independent states. They are indicated by suffixes in the following section.

### I_CaL_ states

Basic formulas of the LTCC:





### Currents













































































## I_to_




















## I_Kur_
















## I_Kr_










## I_Kss_





## I_K1_





## I_KACh_

$$\begin{matrix} g_{KACh\_\max}=6.534\begin{matrix} & \end{matrix}[\text{nS/pF}] \\ \mathrm{n}_{KACh}=1.5 \\ \text{Km}_{KACh}=0.28\begin{matrix} & \end{matrix}[\text{μM}] \\ \mathrm{K}_{ACh}=\frac{[\mathrm{ACh}]^{\mathrm{n}_{KACh}}}{\text{Km}_{KACh}+[\mathrm{ACh}]^{\mathrm{n}_{KACh}}} \end{matrix}$$

$$\begin{matrix} \alpha_{j}=73.1 \\ \beta_{j}=\frac{120}{1+\text{e}^{-(\mathrm{V}\text{+}70)/15}} \\ \alpha_{k}=3.7 \\ \beta_{k}=\frac{5.82}{1+\text{e}^{-(\mathrm{V}\text{+}70)/15}} \end{matrix}$$

$$I_{KACh}=g_{KACh\_\max}\cdot jk\cdot\mathrm{K}_{ACh}\frac{K_{o}}{10+K_{o}}\cdot\frac{V-E_{K}-6.5}{1+0.5\text{e}^{(V-E_{K}-120)\cdot F/2.5RT}}$$

## I_KCa_

$$g_{KCa}=4.4\times10^{-6} V^{3}+4\times10^{-4} V^{2}+0.0164 V+0.7488 \begin{matrix} & \end{matrix}[mS/uF]$$

$$I_{KCa}=g_{KCa}\times(\text{O}1_{KCa}+\text{O}2_{KCa})\times(V-Ek)$$

$$kf1=120\begin{matrix} & \end{matrix}[\text{uM}^{-1}\text{s}^{-1}]$$

$$kf2=96 \begin{matrix} & \end{matrix}[\text{uM}^{-1}\text{s}^{-1}]$$

$$kf3=48 \begin{matrix} & \end{matrix}[\text{uM}^{-1}\text{s}^{-1}]$$

$$kb1=80 \begin{matrix} & \end{matrix}[1/\text{s}]$$

$$kb2=80 \begin{matrix} & \end{matrix}[1/\text{s}]$$

$$kb3=200 \begin{matrix} & \end{matrix}[1/\text{s}]$$

$$kfo1=160 \begin{matrix} & \end{matrix}[1/\text{s}]$$

$$kfo2=1200 \begin{matrix} & \end{matrix}[1/\text{s}]$$

$$kbo1=1000 \begin{matrix} & \end{matrix}[1/\text{s}]$$

$$kbo2=100 \begin{matrix} & \end{matrix}[1/\text{s}]$$

$$C1=1-C2_{KCa}-C3_{KCa}-C4_{KCa}-O1_{KCa}-O2_{KCa}$$

## I_NCX_

















## I_NaK_











## Background currents, I_PMCA_ and I_ClCa_



















# Membrane potential







# SR fluxes

## J_rel_























## J_leak_








## J_up_











# Ion concentration

## Buffers

## Diffusion and ion concentrations

### Na^+^

### Ca^2+^

### K^+^

$$I_{K\_tot}=I_{to}+I_{Kr}+I_{KACh}+I_{K1}+I_{KCa}+I_{Kb}-2 I_{Nak}+I_{Kur}+I_{Kss}+I_{CaK\_junc}+I_{CaK\_sl}$$

### Cl^-^

# References

1. Morotti S, Edwards AG, McCulloch AD, Bers DM, Grandi E. A novel computational model of mouse myocyte electrophysiology to assess the synergy between Na^+^ loading and CaMKII. J Physiol. 2014;592: 1181–1197. doi:10.1113/jphysiol.2013.266676

2. Saucerman JJ, Bers DM. Calmodulin Mediates Differential Sensitivity of CaMKII and Calcineurin to Local Ca^2+^ in Cardiac Myocytes. Biophys J. 2008;95: 4597–4612. doi:10.1529/biophysj.108.128728

3. Soltis AR, Saucerman JJ. Synergy between CaMKII Substrates and β-Adrenergic Signaling in Regulation of Cardiac Myocyte Ca^2+^ Handling. Biophys J. 2010;99: 2038–2047. doi:10.1016/j.bpj.2010.08.016
