# Supplementary material for: A Mathematical Model of the Mouse Atrial Myocyte With Inter-Atrial Electrophysiological Heterogeneity
Source: Front Physiol. 2020 Aug 6;11:972. doi: 10.3389/fphys.2020.00972 (PMC7425199; doi:10.3389/fphys.2020.00972)
Supplement: Supplementary file 3 [file Data_Sheet_3.docx]

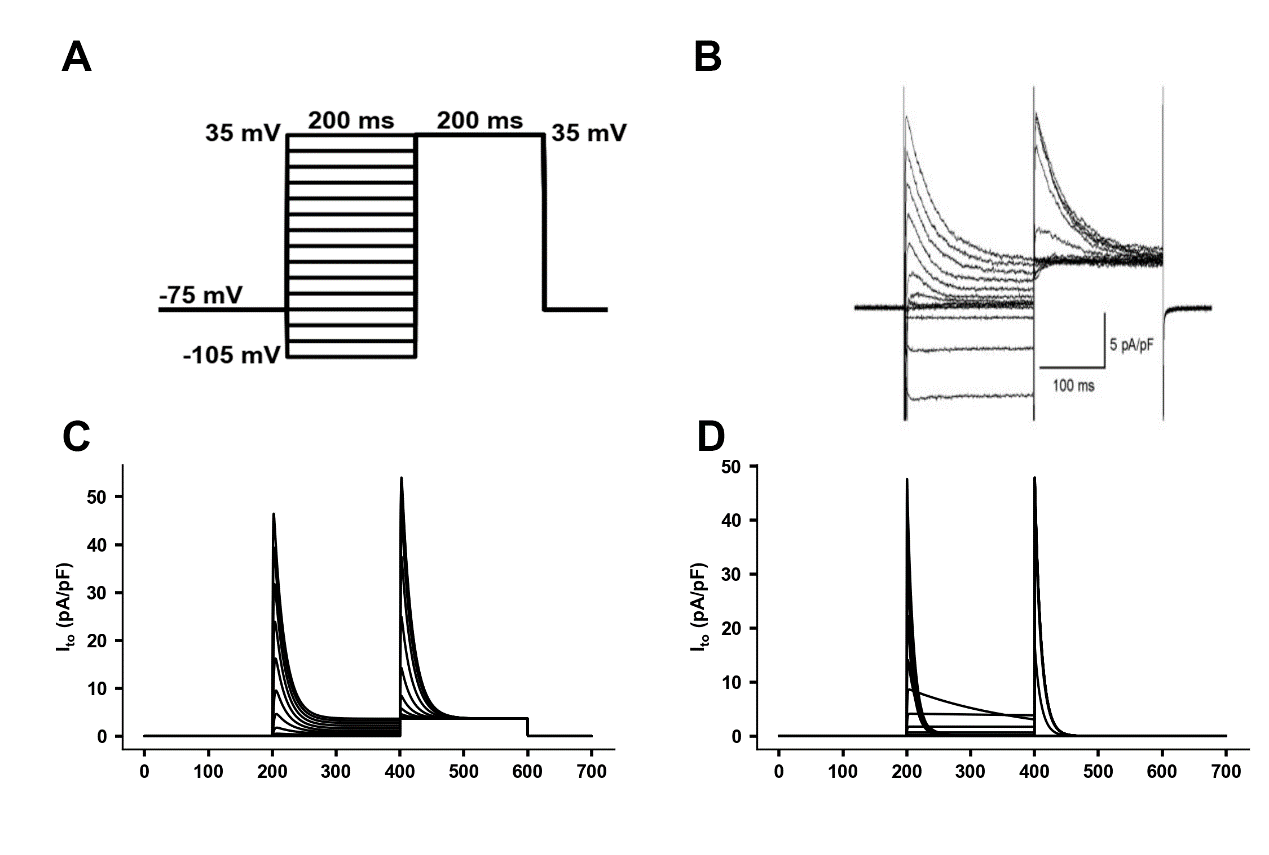


**Supplementary Figure 1. Protocol and current traces for measuring the inactivation of Ito.** (A) Simulated voltage-clamp protocol. (B) Experiment data from Lomax *et al.* at 22°C. (Lomax *et al.*, 2003) (C) Simulation from our Markov chain model, and (D) the Hodgkin & Huxley model (Morotti *et al.*, 2014) of I_to_ at 37°C.

**References**

Lomax, A. E., Kondo, C. S., and Giles, W. R. (2003). Comparison of time- and voltage-dependent K+ currents in myocytes from left and right atria of adult mice. *Am. J. Physiol. - Heart Circ. Physiol.* 285, H1837–H1848. doi:10.1152/ajpheart.00386.2003.

Morotti, S., Edwards, A. G., McCulloch, A. D., Bers, D. M., and Grandi, E. (2014). A novel computational model of mouse myocyte electrophysiology to assess the synergy between Na+ loading and CaMKII. *J. Physiol.* 592, 1181–1197. doi:10.1113/jphysiol.2013.266676.
