# Supplementary material for: A Mathematical Model of the Mouse Atrial Myocyte With Inter-Atrial Electrophysiological Heterogeneity
Source: Front Physiol. 2020 Aug 6;11:972. doi: 10.3389/fphys.2020.00972 (PMC7425199; doi:10.3389/fphys.2020.00972)
Supplement: Supplementary file 4 [file Data_Sheet_4.docx]

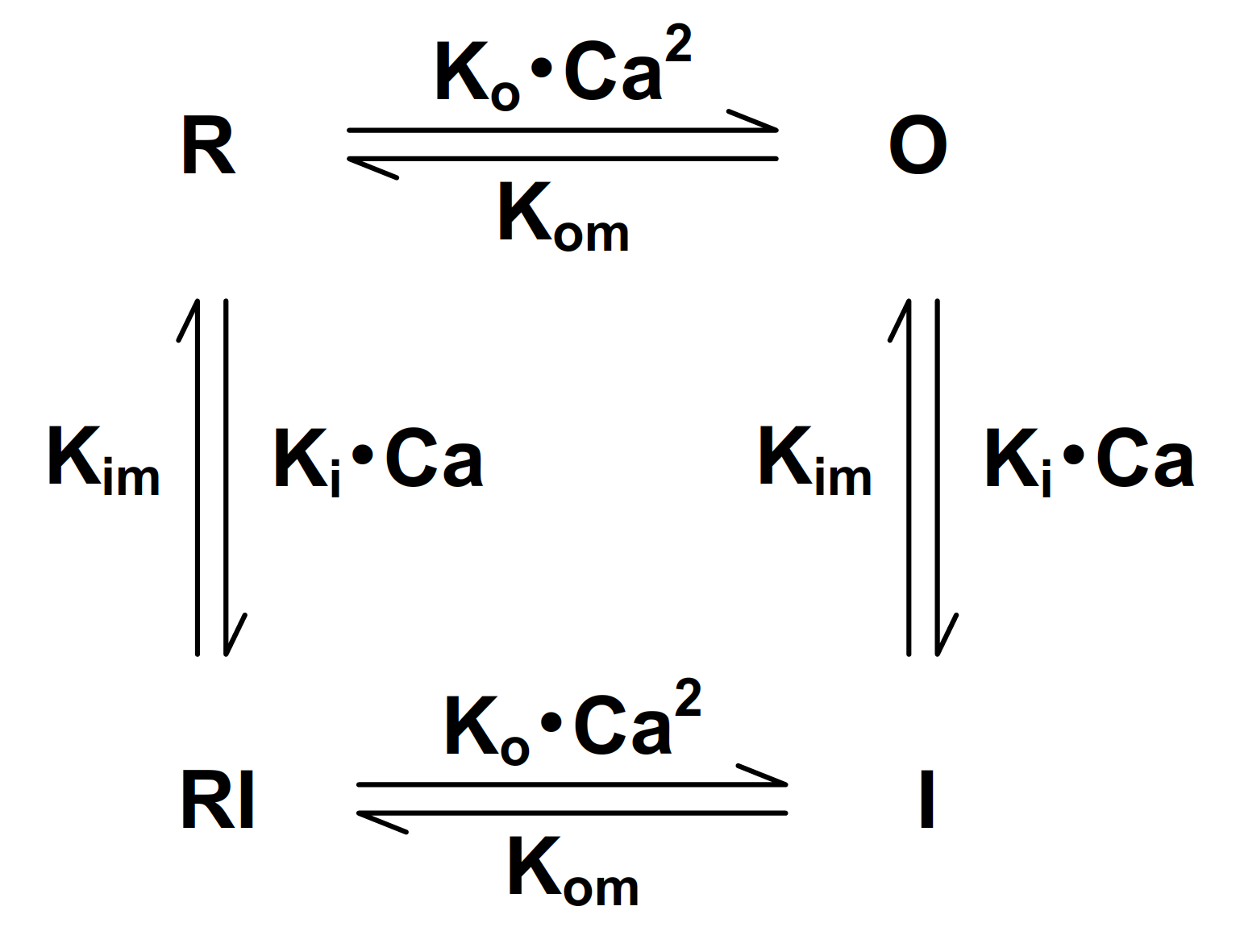

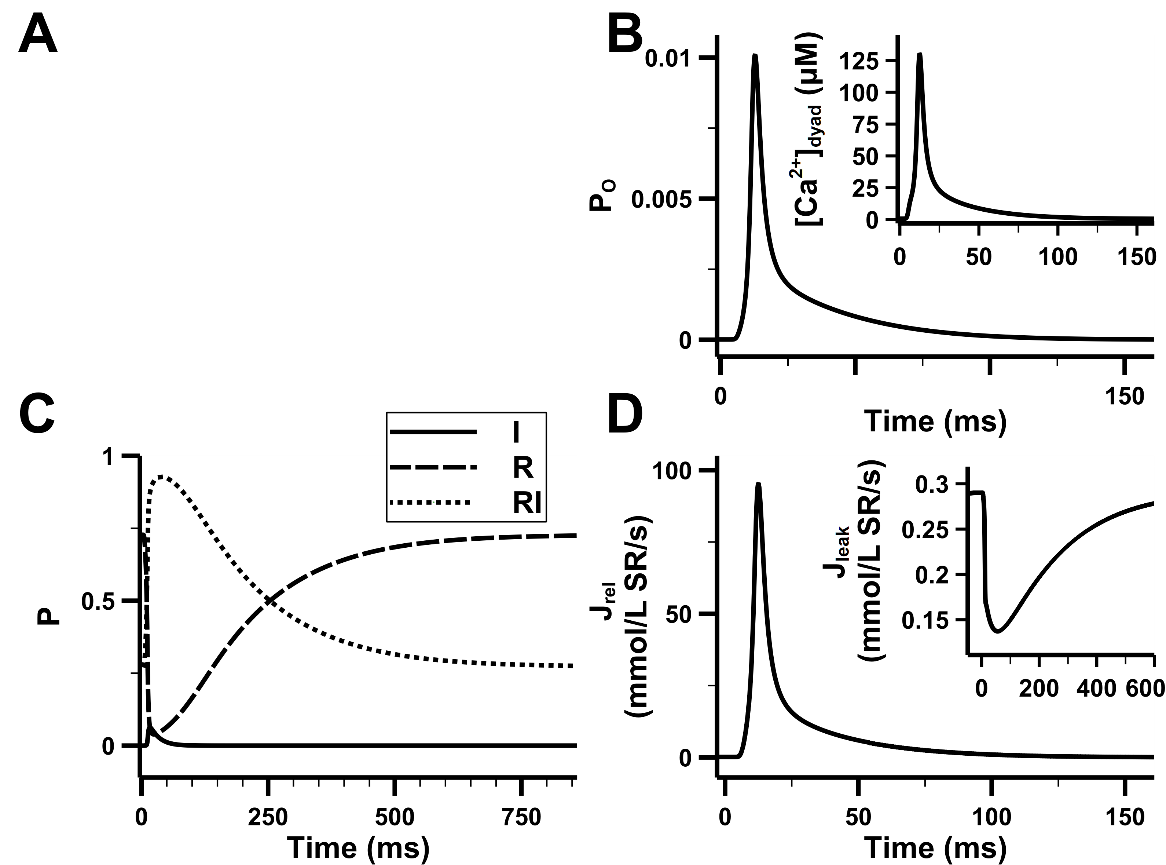


**Supplementary Figure 2. RyR in the mouse atrial model.** (A) Markov chain model of the RyR. (B) Time-dependent profile of the channel open state with CaT in the dyadic cleft (inset). (C) Channel profile of the resting (R), resting inactivated (RI) and inactivated (I) state during an AP. (D) SR Ca^2+^ release flux with RyR-dependent SR Ca^2+^ leak (inset). All states were recorded under 1 Hz pacing rate.
