# Supplementary material for: A Mathematical Model of the Mouse Atrial Myocyte With Inter-Atrial Electrophysiological Heterogeneity
Source: Front Physiol. 2020 Aug 6;11:972. doi: 10.3389/fphys.2020.00972 (PMC7425199; doi:10.3389/fphys.2020.00972)
Supplement: Supplementary file 5 [file Data_Sheet_5.docx]

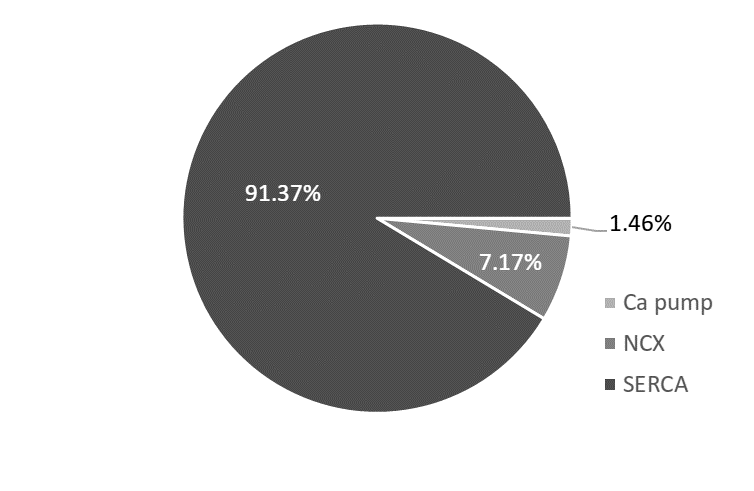

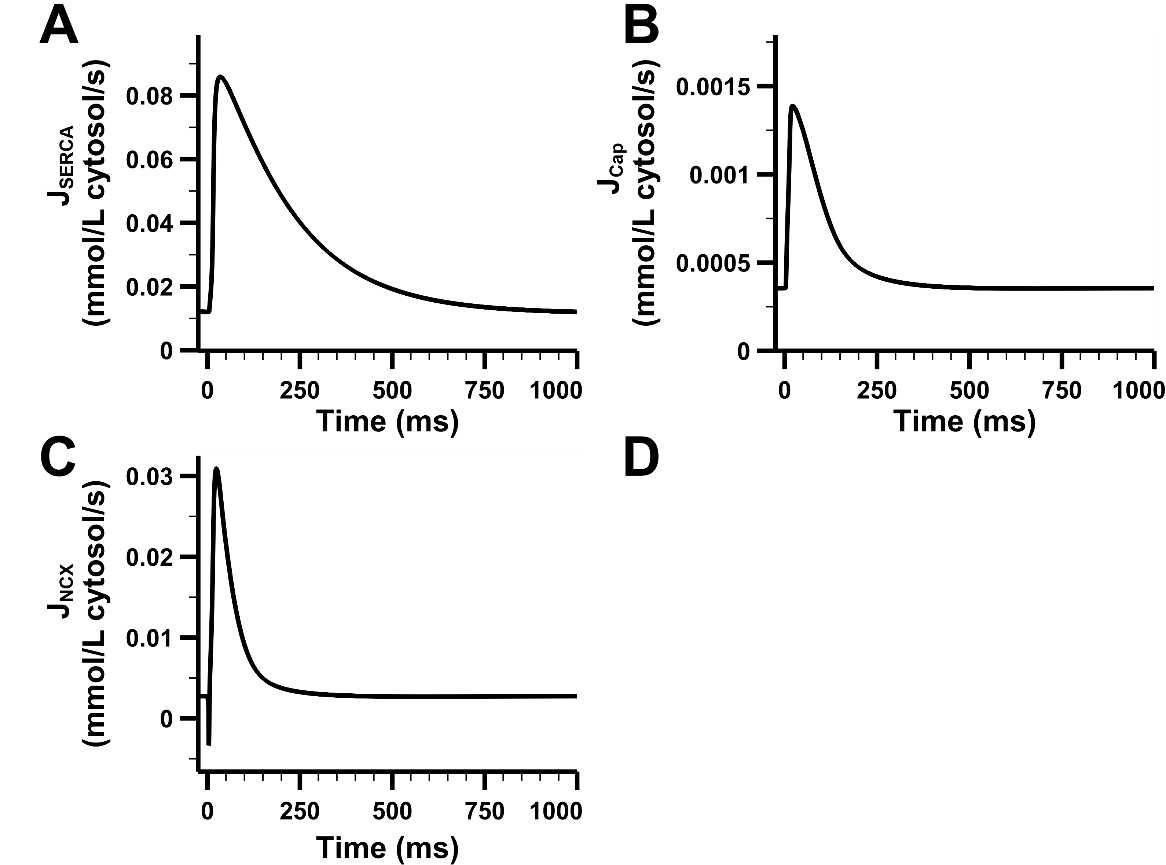


**Supplementary Figure 3.** **Ca^2+^ fluxes during one cardiac cycle in the mouse atrial cell model.** (A) Ca^2+^ extrusion from cytosol by SERCA. (B) Ca^2+^ extrusion from cytosol by sarcolemmal Ca^2+^ pump. (C) Ca^2+^ extrusion from cytosol by NCX. (D) Ca^2+^ removal contributed by SERCA, NCX and Ca^2+^ pump.
