# Supplementary material for: A Mathematical Model of the Mouse Atrial Myocyte With Inter-Atrial Electrophysiological Heterogeneity
Source: Front Physiol. 2020 Aug 6;11:972. doi: 10.3389/fphys.2020.00972 (PMC7425199; doi:10.3389/fphys.2020.00972)
Supplement: Supplementary file 6 [file Data_Sheet_6.docx]

**
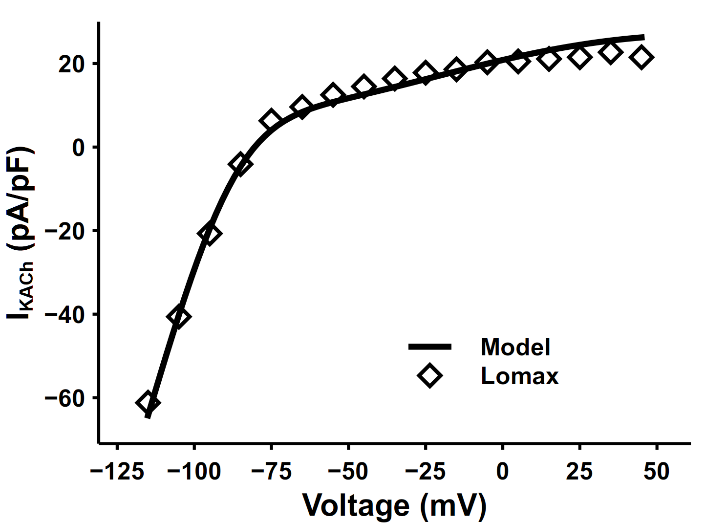
**

**Supplementary Figure 4.** **Simulated and experimental I-V relationship of I_KACh_.** 10 μM acetylcholine was applied to induce the current. The channel was tested at indicated potentials from a holding potential of -75 mV. Experimental data: Lomax *et al.*, (2003).

**Reference**

Lomax, A. E., Rose, R. A., and Giles, W. R. (2003). Electrophysiological evidence for a gradient of G protein-gated K^+^ current in adult mouse atria. *Br. J. Pharmacol.* 140, 576–584. doi:10.1038/sj.bjp.0705474.
