# Supplementary material for: A Mathematical Model of the Mouse Atrial Myocyte With Inter-Atrial Electrophysiological Heterogeneity
Source: Front Physiol. 2020 Aug 6;11:972. doi: 10.3389/fphys.2020.00972 (PMC7425199; doi:10.3389/fphys.2020.00972)
Supplement: Supplementary file 7 [file Table_1.docx]

**Supplementary Table 1. Experimental data for the cycle length (CL), AP amplitude (APA), RP, APD_50_ and APD_90_ under room and body temperature in the mouse atrial myocyte.**

| **Ref** | **CL (ms)** | **RA** | | | | **LA** | | | |
| --- | --- | --- | --- | --- | --- | --- | --- | --- | --- |
|  |  | **APA (mV)** | **RP (mV)** | **APD_50_ (ms)** | **APD_90_ (ms)** | **APA (mV)** | **RP (mV)** | **APD_50_ (ms)** | **APD_90_ (ms)** |
| **Room Temperature** | | | | | | | | | |
| (Lemoine *et al.*, 2011) | 500 |  |  |  |  |  |  | 12 | 85±2 |
| (Qin *et al.*, 2012) | 150 |  | -78.3±1.8 | 12.9±1.1 | 29.7±2.9 |  |  |  |  |
| (Lomax *et al.*, 2003a) | 120 |  | -72.4±1.5 | 10.97±1.77 | 44.03±5.49 |  | -73.5±1.4 | 7.4±0.6 | 27.1±3.3 |
| (Hu *et al.*, 2005) |  |  | -63.2±1.1 |  | 19.00±1.89 (APD80) |  | -64.4±0.8 |  | 12.9±1.7 (APD80) |
| (Reil *et al.*, 2010) |  |  | -70.9±0.9 |  | 24.8±7.3 (APD70) |  | -70.9±1.2 |  | 17.0±3.7 (APD70) |
| (Lomax *et al.*, 2003b) | 100 |  | -68.3±1.4 | 11.3±2.6 | 57.6±7.3 |  | -70.7±7.9 | 8.1±1.6 | 33.2±5.3 |
| (Hirose *et al.*, 2009) | 150 |  |  |  |  |  |  |  | 13.5±1.7 (APD80) |
| (Hua *et al.*, 2012) | 150 |  | -76.9±0.2 | 8.2±0.9 | 45±4.6 |  |  |  |  |
| (Syeda *et al.*, 2016) | 100 |  |  |  |  |  |  | 6.6±0.2 | 19.9±0.9 |
| (Hua *et al.*, 2015) |  | 63.0 ± 5.4 (overshoot) | -81.3 ± 1.5 | 10.9 ± 3.3 | 47.9 ± 6.0 |  |  |  |  |
| **Body Temperature** | | | | | | | | | |
| (Nygren *et al.*, 2004) | 130 |  |  |  | 20.1±0.7 (APD70) |  |  |  | 17.6±0.7 (APD70) |
| (Koh *et al.*, 2001) | 1000 |  |  |  |  | 90.8±4.6 | -78.8±1.6 |  | 90.4±21.8 |
| (Verheule *et al.*, 2004) | 150 | 93.1±3.7 | -77.4±1.1 | 17.4±1.3 (APD60) | 41.8±2.5 | 97.1±4.2 | -77.0±2.6 | 19.2±2.0 (APD60) | 41.8±3.8 |
| (Saegusa *et al.*, 2005) | 200 |  |  |  |  |  |  |  | 25.56 |
| (Bagwe *et al.*, 2005) | 200 | 101±11 | -85±5 | 11.5±3.3 | 34±1.1 |  |  |  |  |
| (Knollmann *et al.*, 2007) | 130 |  |  |  |  |  |  | 9.4±0.4 | 31±1.7 |
| (Odening *et al.*, 2009) |  |  |  |  | 27±8 |  |  |  |  |
| (Nakamura *et al.*, 2010) | 300 | 112.9±1.9 | -82.0±0.8 | 17.6±1.1 | 69.0±4.5 | 117.0±3.3 | –80.7±1.1 | 12.4±0.7 | 53.1±2.4 |
| (Glukhov *et al.*, 2010) | 200 |  |  |  | 33.9±1.8 |  |  |  | 34.0±2.5 |
| (Choi *et al.*, 2012) | 150 |  |  |  | 33.0±8.4 (APD80) |  |  |  | 30.6±7.5 (APD80) |
| (King *et al.*, 2013) | 125 |  |  |  |  | 91.8±6.5 | -75.8±1.4 | 7.38 | 26.4±1.9 |
| (Faggioni *et al.*, 2014) | 100 |  |  |  | 31.2±3.2 |  |  |  | 30.5±1.2 |

**References**

Bagwe, S., Berenfeld, O., Vaidya, D., Morley, G. E., and Jalife, J. (2005). Altered Right Atrial Excitation and Propagation in Connexin40 Knockout Mice. *Circulation* 112, 2245–2253. doi:10.1161/CIRCULATIONAHA.104.527325.

Choi, E.-K., Chang, P.-C., Lee, Y.-S., Lin, S.-F., Zhu, W., Maruyama, M., *et al.* (2012). Triggered Firing and Atrial Fibrillation in Transgenic Mice With Selective Atrial Fibrosis Induced by Overexpression of TGF-β1. *Circ. J. Off. J. Jpn. Circ. Soc.* 76, 1354–1362.

Faggioni, M., Savio-Galimberti, E., Venkataraman, R., Hwang, H. S., Kannankeril, P. J., Darbar, D., *et al.* (2014). Suppression of Spontaneous Ca Elevations Prevents Atrial Fibrillation in Calsequestrin 2-Null Hearts. *Circ. Arrhythm. Electrophysiol.* 7, 313–320. doi:10.1161/CIRCEP.113.000994.

Glukhov, A. V., Flagg, T. P., Fedorov, V. V., Efimov, I. R., and Nichols, C. G. (2010). Differential KATP channel pharmacology in intact mouse heart. *J. Mol. Cell. Cardiol.* 48, 152–160. doi:10.1016/j.yjmcc.2009.08.026.

Hirose, M., Takeishi, Y., Niizeki, T., Shimojo, H., Nakada, T., Kubota, I., *et al.* (2009). Diacylglycerol kinase ζ inhibits Gαq-induced atrial remodeling in transgenic mice. *Heart Rhythm* 6, 78–84. doi:10.1016/j.hrthm.2008.10.018.

Hu, Y., Jones, S. V. P., and Dillmann, W. H. (2005). Effects of hyperthyroidism on delayed rectifier K+ currents in left and right murine atria. *Am. J. Physiol. - Heart Circ. Physiol.* 289, H1448–H1455. doi:10.1152/ajpheart.00828.2004.

Hua, R., Adamczyk, A., Robbins, C., Ray, G., and Rose, R. A. (2012). Distinct Patterns of Constitutive Phosphodiesterase Activity in Mouse Sinoatrial Node and Atrial Myocardium. *PLOS ONE* 7, e47652. doi:10.1371/journal.pone.0047652.

Hua, R., MacLeod, S. L., Polina, I., Moghtadaei, M., Jansen, H. J., Bogachev, O., *et al.* (2015). Effects of Wild-Type and Mutant Forms of Atrial Natriuretic Peptide on Atrial Electrophysiology and Arrhythmogenesis. *Circ. Arrhythm. Electrophysiol.* 8, 1240–1254. doi:10.1161/CIRCEP.115.002896.

King, J. H., Zhang, Y., Lei, M., Grace, A. A., Huang, C. L.-H., and Fraser, J. A. (2013). Atrial arrhythmia, triggering events and conduction abnormalities in isolated murine RyR2-P2328S hearts. *Acta Physiol.* 207, 308–323. doi:10.1111/apha.12006.

Knollmann, B. C., Schober, T., Petersen, A. O., Sirenko, S. G., and Franz, M. R. (2007). Action potential characterization in intact mouse heart: steady-state cycle length dependence and electrical restitution. *Am. J. Physiol. - Heart Circ. Physiol.* 292, H614–H621. doi:10.1152/ajpheart.01085.2005.

Koh, J. T., Choi, H. H., Ahn, K. Y., Kim, J. U., Kim, J. H., Chun, J.-Y., *et al.* (2001). Cardiac Characteristics of Transgenic Mice Overexpressing Refsum Disease Gene-Associated Protein within the Heart. *Biochem. Biophys. Res. Commun.* 286, 1107–1116. doi:10.1006/bbrc.2001.5510.

Lemoine, M. D., Duverger, J. E., Naud, P., Chartier, D., Qi, X. Y., Comtois, P., *et al.* (2011). Arrhythmogenic left atrial cellular electrophysiology in a murine genetic long QT syndrome model. *Cardiovasc. Res.* 92, 67–74. doi:10.1093/cvr/cvr166.

Lomax, A. E., Kondo, C. S., and Giles, W. R. (2003a). Comparison of time- and voltage-dependent K+ currents in myocytes from left and right atria of adult mice. *Am. J. Physiol. - Heart Circ. Physiol.* 285, H1837–H1848. doi:10.1152/ajpheart.00386.2003.

Lomax, A. E., Rose, R. A., and Giles, W. R. (2003b). Electrophysiological evidence for a gradient of G protein-gated K+ current in adult mouse atria. *Br. J. Pharmacol.* 140, 576–584. doi:10.1038/sj.bjp.0705474.

Nakamura, H., Ding, W.-G., Sanada, M., Maeda, K., Kawai, H., Maegawa, H., *et al.* (2010). Presence and functional role of the rapidly activating delayed rectifier K+ current in left and right atria of adult mice. *Eur. J. Pharmacol.* 649, 14–22. doi:10.1016/j.ejphar.2010.08.025.

Nygren, A., Lomax, A. E., and Giles, W. R. (2004). Heterogeneity of action potential durations in isolated mouse left and right atria recorded using voltage-sensitive dye mapping. *Am. J. Physiol. - Heart Circ. Physiol.* 287, H2634–H2643. doi:10.1152/ajpheart.00380.2004.

Odening, K. E., Nerbonne, J. M., Bode, C., Zehender, M., and Brunner, M. (2009). In Vivo Effect of a Dominant Negative Kv4.2 Loss-of-Function Mutation Eliminating I_to,f_ on Atrial Refractoriness and Atrial Fibrillation in Mice. *Circ. J.* 73, 461–467. doi:10.1253/circj.CJ-08-0840.

Qin, M., Huang, H., Wang, T., Hu, H., Liu, Y., Gu, Y., *et al.* (2012). Atrial Tachyarrhythmia in Rgs5-Null Mice. *PLOS ONE* 7, e46856. doi:10.1371/journal.pone.0046856.

Reil, J.-C., Hohl, M., Oberhofer, M., Kazakov, A., Kaestner, L., Mueller, P., *et al.* (2010). Cardiac Rac1 overexpression in mice creates a substrate for atrial arrhythmias characterized by structural remodelling. *Cardiovasc. Res.*, cvq079. doi:10.1093/cvr/cvq079.

Saegusa, N., Sato, T., Saito, T., Tamagawa, M., Komuro, I., and Nakaya, H. (2005). Kir6.2-deficient mice are susceptible to stimulated ANP secretion: KATP channel acts as a negative feedback mechanism? *Cardiovasc. Res.* 67, 60–68. doi:10.1016/j.cardiores.2005.03.011.

Syeda, F., Holmes, A. P., Yu, T. Y., Tull, S., Kuhlmann, S. M., Pavlovic, D., *et al.* (2016). PITX2 Modulates Atrial Membrane Potential and the Antiarrhythmic Effects of Sodium-Channel Blockers. *J. Am. Coll. Cardiol.* 68, 1881–1894. doi:10.1016/j.jacc.2016.07.766.

Verheule, S., Sato, T., Everett, T., Engle, S. K., Otten, D., Lohe, M. R. der, *et al.* (2004). Increased Vulnerability to Atrial Fibrillation in Transgenic Mice With Selective Atrial Fibrosis Caused by Overexpression of TGF-β1. *Circ. Res.* 94, 1458–1465. doi:10.1161/01.RES.0000129579.59664.9d.
